# Strongly visible light-absorbing metal–organic frameworks functionalized by cyclometalated ruthenium(ii) complexes†

**DOI:** 10.1039/c9ra06984d

**Published:** 2020-03-02

**Authors:** Eirik Mydske Thoresen, Sigurd Øien-Ødegaard, Gurpreet Kaur, Mats Tilset, Karl Petter Lillerud, Mohamed Amedjkouh

**Affiliations:** Department of Chemistry, University of Oslo P. O. Box 1033, Blindern 0315 Oslo Norway mamou@kjemi.uio.no +47-22857009; Center for Materials Science and Nanotechnology (SMN), Faculty of Mathematics and Natural Sciences, University of Oslo P. O. Box 1126, Blindern 0318 Oslo Norway

## Abstract

Four different ruthenium(ii) complexes were incorporated into the metal–organic framework (MOF) UiO-67 using three different synthetic strategies: premade linker synthesis, postsynthetic functionalization, and postsynthetic linker exchange. One of these complexes was of the type (N–N)_3_Ru^2+^, and three of the complexes were of the type (N–N)_2_(N–C)Ru^+^, where N–N is a bipyridine-type ligand and N–C is a cyclometalated phenylpyridine-type ligand. The resulting materials were characterized by PXRD, SC-XRD (the postsynthetic functionalization MOFs), N_2_ sorption, TGA-DSC, SEM, EDS, and UV-Vis spectroscopy, and were digested in base for subsequent ^1^H NMR analysis. The absorption profiles of the MOFs that were functionalized with cyclometalated Ru(ii) complexes extend significantly further into the visible region of the spectrum compared to the absorption profiles of the MOFs that were functionalized with the non-cyclometalated reference, (N–N)_3_Ru^2+^.

## Abstract 2

The metal–organic framework (MOF) UiO-67 was functionalized by incorporating different cyclometalated ruthenium(ii) complexes using three different methods: premade linker synthesis, postsynthetic functionalization, and postsynthetic linker exchange.

## Graphical abstract

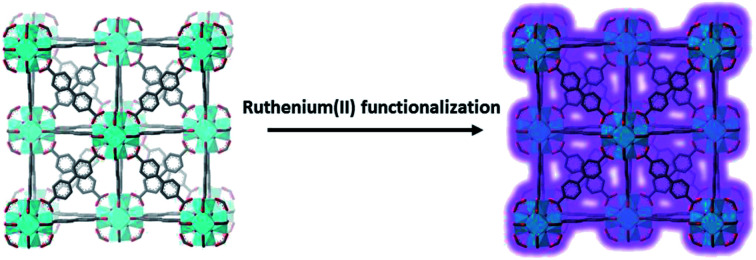

## Introduction

Metal–organic frameworks (MOFs) have gained increasing interest in recent years due to the versatility these materials have shown in applications, and the potential they represent for the future development. These applications include gas storage^1–3^ and separation,^4–6^ catalysis^7–10^ and sensors,^11,12^ among others.^13^ Photocatalysis using MOFs has emerged as an important field of research, and for such applications, photosensitizers are usually required.^7,14–17^ These are molecules that, upon excitation by light, produce a chemical or physical change in another species. They may be present as free molecules in solution (filling the pores of the MOF material), or may be integrated as part of the MOF structure. Ruthenium(ii) complexes with bipyridine (bpy) derived ligands, which are known for their intense metal–ligand charge transfer (MLCT) transitions, constitute a class of photosensitizers that have been studied in both of these settings.^16,18–26^ Another field of photochemistry in which Ru(ii) complexes play a central role is their use in dye sensitized solar cells (DSSCs).^27,28^ Here, the replacement of N–N (bpy-type) ligands with structurally analogous N–C ligands (cyclometalated phenylpyridines) has successfully provided enhanced absorption of visible light. This property is largely caused by the decrease of the HOMO–LUMO energy gaps and the lowering of the molecular symmetries when the cyclometalated ligands are introduced.^29–31^

The inclusion of cyclometalated Ru(ii) complexes in MOFs should cause the absorption bands of these materials to extend significantly further into the visible region of the spectrum, compared to previous reports where MOFs have been functionalized with amino groups on the aromatic linkers,^32–34^ or with the non-cyclometalated Ru(bpy)_3_ moiety (complex 1).^16,18,19,21,24^

**Fig. 1:**
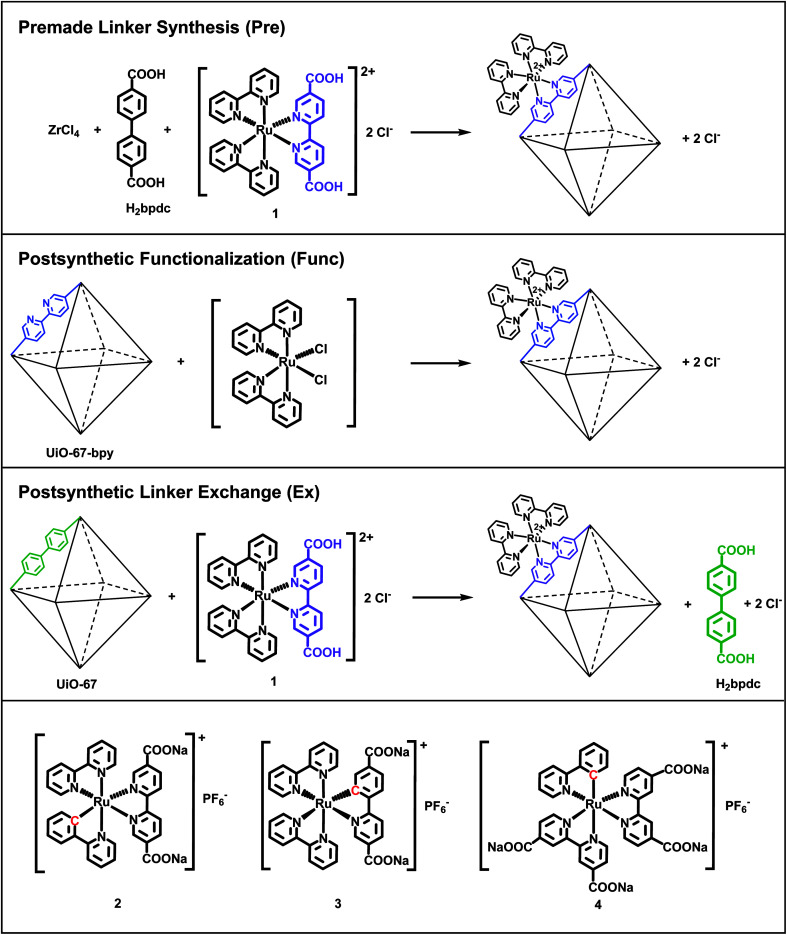
The three functionalization methods used in this work to incorporate Ru(ii) complexes into UiO-67, illustrated with complex 1. The three cyclometalated Ru(ii) complexes (2, 3 and 4) are shown in the bottom row. The octahedra represent the octahedral cages in UiO-67.

In this work, we have incorporated cyclometalated Ru(ii) complexes 2, 3 and 4 (Fig. 1) as linkers into the MOF UiO-67. This Zr-based MOF is known for its high chemical and thermal stability.^35^ The current contribution is a continuation of our previous report on the synthesis, characterization, and computational investigation of the Ru(ii) complexes 1–4 in Fig. 1.^36^ Complex 1, which has become a standard integrated photosensitizer in MOFs,^16,18,19,21,24^ was chosen in this study as a non-cyclometalated reference. Complex 4 is known as a sensitizer in DSSCs.^29,31,37,38^

Complexes 1–4 are here attempted incorporated into UiO-67 using three different synthetic protocols: premade linker synthesis,^16,18,39,40^ postsynthetic functionalization,^20,21,41–44^ and postsynthetic linker exchange^23,45–48^ (Fig. 1), all of which are methods that have been described in the literature for other systems.

The combination of the four Ru(ii) complexes 1–4 and three functionalization methods in this article has resulted in ten different synthesized MOF systems. Each Ru(ii)-functionalized MOF system is named after the Ru(ii) complex (1–4) and the functionalization method (premade linker synthesis = Pre, postsynthetic functionalization = Func, and postsynthetic linker exchange = Ex). Complexes 3 and 4 cannot be incorporated by the postsynthetic functionalization method. Complex 3 is synthesized *via* base promoted C–H activation,^36^ which is not feasible for reaction with the MOF structure. UiO-67 does not contain linkers that can be substituted for any of the ligands in complex 4, as will be discussed later.

## Experimental section

### General

All chemicals were used as received. UiO-67,^35,49^ UiO-67-bpy,^41^ single crystals of UiO-67-Me_2_-bpy,^50^ and all ruthenium complexes^36,51^ were synthesized as previously reported. Centrifugation of samples was done using 15 mL centrifuge tubes (60 mL sample glass for the first centrifugation of the postsynthetic linker exchange samples) and a Heraeus Labofuge 400 instrument from Thermo Scientific at 3000 rpm for 10 min. Washing of the samples was performed by shaking the powder dispersed in the solvent in a centrifuge tube on an IKA KS260 shaker at 300 rpm for 15 min, followed by centrifugation and decantation.

### Synthesis of 1-Pre

ZrCl_4_ (91 mg, 0.39 mmol, 1 equiv.) was dissolved in DMF (10 mL) in a 25 mL beaker before water (0.021 mL, 1.17 mmol, 3 equiv.) was added. Benzoic acid (476 mg, 3.9 mmol, 10 equiv.) was added as a modulator and dissolved before biphenyl-4,4′-dicarboxylic acid (H_2_bpdc, 85 mg, 0.35 mmol, 0.9 equiv.) was added and dissolved by heating and stirring on a hotplate for a couple of minutes. Heating was discontinued before complex 1 (28 mg, 0.04 mmol, 0.1 equiv.) was added as the functionalized linker to the still hot solution and dissolved. The solution was then poured over in a preheated 25 mL Erlenmeyer flask, then capped and heated without stirring in an oven at 120 °C for three days. After reaction, the mixture was cooled to room temperature, centrifuged and decanted. Subsequently the powder was washed with DMF (3 × 7 mL) and methanol (3 × 7 mL) before it was dried at 100 °C in air overnight to yield an orange powder (128 mg).

### Synthesis of 2-Pre

Following the same procedure as for 1-Pre, complex 2 (33 mg, 0.04 mmol, 0.1 equiv.) was used as the functionalized linker. This gave the product as a grey powder (139 mg).

### Synthesis of 3-Pre

Following the same procedure as for 1-Pre, complex 3 (35 mg, 0.04 mmol, 0.1 equiv.) was used as the functionalized linker. This gave the product as a white powder (107 mg).

### Synthesis of 4-Pre

Following the same procedure as for 1-Pre, complex 4 (38 mg, 0.04 mmol, 0.1 equiv.) was used as the functionalized linker. This gave the product as a dark purple powder (122 mg).

### Synthesis of 1-Func

UiO-67-bpy-5%^37^ (133 mg, containing 0.0031 mmol bpydc linkers, 1 equiv.) was mixed with *cis*-Ru(bpy)_2_Cl_2_ (8 mg, 0.016 mmol, 5 equiv.) and EtOH (7 mL) in a 25 mL round bottom flask. The mixture was stirred at reflux for 21 hours. After reaction, the mixture was centrifuged and decanted. Subsequently the powder was washed with dichloromethane (3 × 7 mL) before it was dried at 100 °C in air overnight to yield a light orange/brown powder (123 mg).

### Synthesis of 1-Func single crystals

Ru(bpy)_2_Cl_2_ (3 mg, 0.006 mmol) was dissolved in MeOH (2 mL) and added to a 4 mL glass vial containing a couple of mg of single crystals of UiO-67-Me_2_-bpy-10%. The vial was closed and the mixture was heated without stirring at 60 °C for three days. The crystals were then isolated, washed with dichloromethane and stored in acetone until SC-XRD measurements were performed.

### Synthesis of 2-Func

The same procedure as for 1-Func was used, except that the Ru(ii) complex was *cis*-[Ru(ppy)(bpy)(MeCN)_2_]PF_6_ (10 mg, 0.016 mmol, 5 equiv.; ppy = 2-phenylpyridine, bpy = 2,2′-bipyridine). This gave the product as a purplish grey powder (128 mg).

### Synthesis of 2-Func single crystals

The same procedure as for 1-Func single crystals was used, except that the Ru(ii) complex was *cis*-[Ru(ppy)(bpy)(MeCN)_2_]PF_6_ (3 mg, 0.005 mmol).

### Synthesis of 1-Ex

Complex 1 (291 mg, 0.4 mmol, 1 equiv.) was dissolved in a mixture of DMF and water (40 mL, 50 : 50) in a 100 mL round bottom flask. UiO-67 (141 mg, 0.4 mmol linkers, 1 equiv.) was added to the solution and the mixture was stirred at 100 °C for three days. After reaction, the mixture was centrifuged and decanted. Subsequently the powder was washed with DMF (3 × 15 mL) and methanol (3 × 15 mL) before it was dried at 100 °C in air overnight to yield an orange powder (112 mg).

### Synthesis of 2-Ex

Following the same procedure as for 1-Ex, complex 2 (338 mg, 0.4 mmol, 1 equiv.) was used as the functionalized linker. 1 M HCl (0.8 mL, 0.8 mmol, 2 equiv.) was also added after dissolution of complex 2 in order to protonate the carboxylate groups. This gave the product as a dark purple powder (119 mg).

### Synthesis of 3-Ex

Following the same procedure as for 1-Ex, complex 3 (360 mg, 0.38 mmol, 1 equiv.) was used as the functionalized linker. 1 M HCl (1.63 mL, 1.63 mmol, 4.3 equiv.) was also added after dissolution of complex 3 in order to protonate the carboxylate groups. This gave the product as a dark maroon powder (151 mg).

### Synthesis of 4-Ex

Following the same procedure as for 1-Ex, complex 4 (391 mg, 0.4 mmol, 1 equiv.) was used as the functionalized linker. 1 M HCl (1.6 mL, 1.6 mmol, 4 equiv.) was also added after dissolution of complex 4 in order to protonate the carboxylate groups. This gave the product as a dark purple powder (92 mg).

### Powder X-ray diffraction (PXRD)

PXRD patterns were collected on a Bruker D8 Discovery diffractometer equipped with a focusing Ge-monochromator, using Cu-Kα_1_ radiation (*λ* = 1.5418 Å) and a Bruker LYNXEYE detector. Patterns were collected in reflectance Bragg–Brentano geometry over a 2*θ* range of 2–50°.

### Single crystal X-ray diffraction (SC-XRD)

Complete data sets for single crystals of 1-Func and 2-Func were acquired on a Bruker D8 venture diffractometer equipped with a photon 100 detector and using Mo Kα radiation (*λ* = 0.71073 Å).

Data reduction was performed with the Bruker Apex3 Suite, the structure was solved with ShelxT^52^ and refined with ShelxL.^53^ Olex2 was used as user interface and to produce Fourier map illustrations.^54^ The occupancy coefficient of Ru(ii) was allowed to refine freely.

The cif files were edited with enCIFer v1.6.^55^

### Nitrogen sorption isotherms

Nitrogen sorption measurements were performed on a BelSorp mini II instrument at 77 K. Prior to measurement, the samples (typically 40–50 mg) were heated under vacuum at 80 °C for 1 h, then at 180 °C for 2 h, in order to remove solvent from the MOF pores.

### Thermogravimetric analysis-differential scanning calorimetry (TGA-DSC)

TGA-DSC measurements were made with a Stanton Redcroft 1500 or a Netzsch 449 F3-Jupiter TGA-DSC instrument, in which 7–20 mg of MOF sample was loaded in a platinum or alumina crucible. Samples were heated from RT to 800 °C at a rate of 5 °C min^−1^ under a constant and simultaneous flow of both N_2_ (20 mL min^−1^) and O_2_ (5 mL min^−1^).

### Scanning electron microscopy (SEM) and energy dispersive X-ray spectroscopy (EDS)

SEM images were obtained with a Hitachi SU8230 field emission scanning electron microscope. The acceleration voltage was set to 2.5 kV and the probe current to 10 μA. In order to reduce sample charging, 1.5 kV deceleration voltage was applied, resulting in an effective voltage (“landing voltage”) of 2.5–1.5 = 1 kV. The same instrument was equipped with a Bruker EDS detector. Samples were prepared as powders or pellets attached to carbon tape. The working distance was 15 mm for EDS analysis, and the scanned area was *ca.* 1000 μm^2^. The accelerating voltage was set to 10 kV so that both zirconium (Lα = 2.042 keV) and ruthenium (Lα = 2.558 keV) could be reliably quantified.

### ^1^H nuclear magnetic resonance (^1^H NMR) of digested MOFs

Samples were prepared by weighing 20 mg of MOF into a centrifuge tube and adding 1 mL of 1 M NaOH in D_2_O. The tube was capped and the mixture was shaken on an IKA KS260 instrument at 300 rpm for 30 min before it was left to digest for 24 hours. The mixture was centrifuged at 3000 rpm for 30 min with a Heraeus Labofuge 400 instrument from Thermo Scientific before the 600 μL of top solution was transferred into an NMR tube. In order to provide more informative spectra, the D_2_O solution was evaporated to dryness with a rotavapor before the solid was dissolved in CD_3_OD. Solution ^1^H NMR spectra were recorded with a Bruker AVIII400 NMR spectrometer (400 MHz).

### Diffuse reflectance UV-visible (DR UV-Vis) spectroscopy

The samples were prepared by filling a circular plastic container (9 mm diameter, 3 mm depth) with MOF powder and flattening the surface with a microscope slide. DR UV-Vis spectra were obtained using a DH-2000 UV-Vis-NIR light source (operating with a deuterium lamp) from Mikropack and a USB2000+ spectrometer from Ocean Optics. BaSO_4_ was used as a reference. The absorbance was calculated from the obtained reflectance data using the Kubelka–Munk theory, which is implemented in the software SpectraSuite from Ocean Optics. For the absorbance of the homogeneous complexes 1–4 in methanol solution, a UV-3600 spectrometer from Shimadzu was employed. Further details of these measurements can be found in our recently published article.^36^

## Results and discussion

As mentioned in the introduction, complexes 1–4 are in this work attempted incorporated into the MOF UiO-67 using three different synthetic methods: premade linker synthesis,^16,18,39,40^ postsynthetic functionalization,^20,21,41–44^ and postsynthetic linker exchange^23,45–48^ (Fig. 1).

In the premade linker synthesis method, ZrCl_4_, biphenyl-4,4′-dicarboxylic acid (H_2_bpdc, the standard, non-functionalized linker), and the Ru(ii) complex (the functionalized linker) are dissolved together before the MOF assembly is initiated. Often, a monocarboxylic acid (a modulator) is added to the reaction mixture in order to slow down the crystallization process, thereby enhancing the crystallinity of the MOF product.^49,56^ In the postsynthetic functionalization method, a MOF in which a fraction of its standard linkers have been replaced with analogues that are capable of coordinating a metal complex (*e.g.* 2,2′-bipyridine-5,5′-dicarboxylic acid, H_2_bpydc, is used in part instead of H_2_bpdc) reacts with a dissolved Ru(ii) precursor, resulting in the Ru(ii)-functionalized MOF. Finally, in the postsynthetic linker exchange method, an unfunctionalized MOF, *i.e.* UiO-67, is suspended in a solution of the Ru(ii)-functionalized linker, which undergoes exchange with the standard linker in the MOF structure. Notably, complex 1 has previously been incorporated into UiO-67 by all these three methods by Yu and Cohen.^19^

In the following, the results are presented and discussed, grouped according to the type of Ru(ii) complex (1–4) used for the preparation of the Ru(ii)-functionalized MOFs. For convenience, selected characterization data are summarized in Table 1.

**Table 1:** Characteristics of the MOFs synthesized in this study. Pictures of the powders, adsorption/desorption isotherms, TGA-DSC traces and EDS spectra are provided in the ESI^a^

MOF	Color	*A*_BET_ (m^2^ g^−1^)	TGA, mass loss range (°C)	SEM, crystallite sizes (μm)	EDS, Ru : Zr ratio
UiO-67	White	2457	480–550	0.5–2	0
UiO-67-bpy (5%)^41^	White	2460	475–540	0.5–2	0
1-Pre	Orange	2300	370–420	0.5–2	0.07 (0.02)
1-Func	Light orange/brown	2457	380–420	0.5–2	0.02 (0.01)
1-Ex	Orange	1694	365–415	0.5–2	0.02 (0.01)
2-Pre	Grey	1996	370–420	0.2–0.5	0.08 (0.01)
2-Func	Grey/purple	2346	360–460	0.5–2	0.05 (0.02)
2-Ex	Dark purple	940	330–390	0.5–1.5	0.18 (0.01)
3-Pre	White	2541	450–520	0.2–0.5	0.01 (0.01)
3-Ex	Dark maroon	1405	365–415	1–2	0.20 (0.01)
4-Pre	Dark purple	1530	370–420	0.75–3	0.16 (0.01)
4-Ex	Dark purple	761	280–355	0.5–2	0.10 (0.01)

a The samples UiO-67 and UiO-67-bpy (5%) in the table are the same samples that were used for the postsynthetic linker exchange (Ex) and postsynthetic functionalization (Func) processes, respectively.

### Incorporation of complex 1

The non-cyclometalated reference complex 1 has previously been incorporated into UiO-67 with all three methods: premade linker synthesis,^16,18,19,24^ postsynthetic functionalization,^19^ and postsynthetic linker exchange.^19^ In this work, all of these were also successful in incorporating complex 1, leading to Ru(ii)-functionalized MOFs 1-Pre, 1-Func, and 1-Ex, respectively. All these MOFs had an orange color, although 1-Func was paler than the other two (Fig. S1†). Analysis of the products by PXRD showed that all MOFs had the intact crystallinity of UiO-67 (Fig. S5†). Nitrogen sorption measurements and BET analysis revealed that the materials were porous, with BET surface areas of 2300 (1-Pre), 2457 (1-Func), and 1694 (1-Ex) m^2^ g^−1^, as can be read from Table 1. The higher surface area of 1-Func compared to 1-Pre could stem from the fact that 1-Func is obtained starting from a pure UiO-67-bpy MOF with higher surface area (2460 m^2^ g^−1^) than 1-Pre. The reactant *cis*-Ru(bpy)_2_Cl_2_ will (ideally) only coordinate at the bpy linkers in the MOF while the excess will be removed during the washing procedure. On the other hand, 1-Pre is synthesized by a one pot method which could lead to some Ru-functionalized linker being trapped in the pores because of the coordinating carboxylate groups. 1-Ex has the lowest surface area of the three MOFs, which could be explained by partial degradation of the structure during the linker exchange process. The corresponding isotherms are shown in Fig. S6–S8,† and are for all the MOFs close to an ideal type I isotherm, with 1-Ex having the largest deviation.

The thermal stabilities of the MOFs in air were probed by TGA (Fig. S16†). The mass loss corresponding to burning of the organic linkers appeared for all MOFs around 420 °C (Table 1). This is approximately 100 °C lower than for pristine UiO-67, probably due to increased strain and defectivity introduced by the Ru(ii) complexes and the synthesis conditions. If the end-weight in a TGA experiment is normalized to 100%, the weight before the mass loss would for pristine UiO-67 be positioned at 282%. For UiO-67 in which all of the linkers are substituted by complex 1 (hypothetical), the corresponding weight would be at 298%, which is 1.06 times higher. For 10% Ru substituted UiO-67, it would be only 1.01 times higher. Thus, the incorporation of Ru(ii) complexes into UiO-67 will not have a significant effect on the relative mass loss in the TGA experiments. However, it is observed that the relative mass loss for 1-Func is *ca.* 10% lower than that for UiO-67-bpy, and that the mass loss for 1-Ex is *ca.* 40% lower than that for UiO-67. These differences can arise from a lower content of organic constituents in the Ru(ii)-functionalized MOFs, *i.e.* missing linker defects.^57,58^ Such defects could be introduced by the postsynthetic treatments of the MOFs, in particular during the linker exchange reaction to obtain 1-Ex, which were designed to partly break the bonds between the clusters and the linkers.

The SEM pictures (Fig. S20†) show that all MOFs consist of octahedral crystallites in the size ranges 0.5–2 μm (Table 1). EDS was employed to obtain estimates of the Ru : Zr ratio in the materials (in ideal UiO-67 there is one Zr atom per linker). The analysis revealed Ru : Zr ratios of 0.07 (1-Pre), 0.02 (1-Func), and 0.02 (1-Ex), as listed in Table 1. Spectra are shown in the ESI (Fig. S23–S29†). In the synthesis of 1-Pre, 10% of the linkers used were Ru(ii)-functionalized, thus the Ru : Zr ratio is within expected values. The Ru : Zr ratio in 1-Func is also not surprising, since a UiO-67 sample with 5% bpydc linkers was used in the functionalization process. Furthermore, these Ru : Zr ratios could partially explain the differences in surface areas between 1-Pre and 1-Func, since the Ru moieties would occupy otherwise free pore volume in the MOFs.

For comparison to the previous reports on the same materials (although they were synthesized with variations in the detailed experimental parameters), 1-Pre has been reported with Ru : Zr ratios of 0.015 (two different studies, using ICP-MS^16^ and NMR^19^ as quantification methods, respectively) and 0.0086 (with the use of UV-Vis spectroscopy, by digesting the MOF and subsequently recording the optical densities at 448 nm of the solution in order to quantify the concentration of the Ru(ii) complex).^18^1-Func has been reported with a Ru : Zr ratio as high as 0.15, in which case UiO-67 with as much as 25% bpydc was used for the synthesis.^19^ In the same work, 1-Ex was reported with a Ru : Zr ratio of 0.01.^19^

The synthesized MOFs were digested in 1 M NaOH in D_2_O for 24 hours before the resulting solutions were analysed by ^1^H NMR. The signals belonging to complex 1 were clearly seen in the spectra of the digested MOFs, indicating that the complex remained intact during the MOF syntheses (Fig. S30–S32†). In the case of 1-Func, this is evidence of proper chemical incorporation of complex 1 as a linker in the MOF structure. This is because *cis*-Ru(bpy)_2_Cl_2_ was used as a reagent instead of complex 1 in the postsynthetic functionalization of the MOF.

**Fig. 2:**
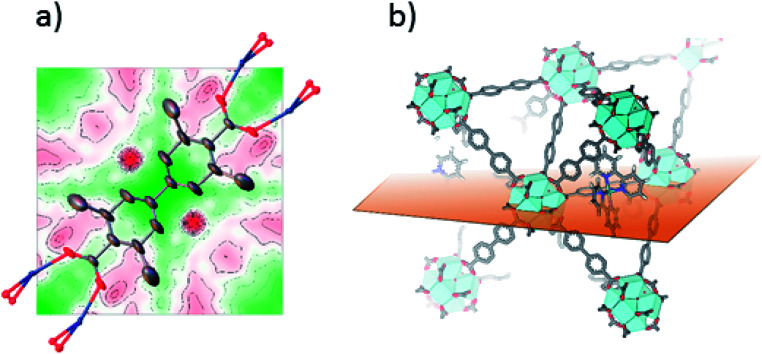
(a) 2D Fourier difference map of 1-Func, parallel to Miller plane 1 0 0, intersecting the expected position of Ru before including it in the crystal structure refinement. Ru is disordered over two symmetry equivalent positions on each side of the linker's axis of connectivity. (b) Representation of the plane used for the Fourier map in the structure of Ru-functionalized UiO-67-Me_2_-bpy.

Single crystals suitable for SC-XRD were also prepared using the postsynthetic functionalization method. In these MOF crystals, 3,3′-dimethylbiphenyl-4,4′-dicarboxylic acid was used as standard linker (instead of unsubstituted biphenyl dicarboxylic acid) in order to obtain single crystals large enough for measurement on a laboratory source. The fraction of the linkers that were substituted by bpydc was 10%. In contrast to the postsynthetic functionalization reactions on powders, the postsynthetic functionalization on single crystals was done in methanol at 60 °C (without stirring) for three days. The structure solution and refinement clearly showed the structure of UiO-67-Me as previously published.^50^ Trying to refine the structure without ruthenium clearly revealed the presence of electron density close to the center of the linker, on the expected location of Ru coordinated to a bipyridine linker. Fig. 2a shows the Fourier difference map (the disagreement between the model and raw data) for 1-Func before Ru was included in the model. The electron density appears on both sides of the linker as these sites are equal by symmetry and Ru can occupy either of them. The occupancy coefficient of Ru refined freely to 7%, which is in agreement with the amount of bpydc in the MOF. This is, in addition to the NMR result, direct evidence for the chemical incorporation of complex 1 as linker in UiO-67.

### Incorporation of complex 2

This cyclometalated Ru(ii) complex was shown in our previous report to have significant absorption in the visible region, and that the corresponding MLCT transitions populate the ligands with the carboxylate groups.^36^ The three MOF functionalization methods led to Ru(ii)-functionalized MOFs 2-Pre, 2-Func, and 2-Ex. As powders, 2-Pre had a grey appearance, 2-Func was greyish, but with a clear hint of purple, and 2-Ex was dark purple (Fig. S2†). 2-Pre were also synthesized with some (independent) variations in the procedure: (1) heating at 90 °C, (2) stirring while heating, and (3) without the use of modulator. None of the variations caused discernible changes in the appearance and color of the MOF. PXRD showed that the MOFs had the crystal structure of UiO-67 (Fig. S5†). The BET surface areas were measured to be 1996 (2-Pre), 2346 (2-Func), and 940 (2-Ex) m^2^ g^−1^. The relative order of these three values is the same as for the corresponding MOFs functionalized with complex 1 (see above) and could be interpreted using the same arguments. 2-Pre shows an adsorption/desorption isotherm with type II characteristics in the high-pressure region, which indicates multilayer formation or condensation (Fig. S9†). 2-Func shows more typical type I behavior (Fig. S10†). The isotherm for 2-Ex (Fig. S11†) shows a distinct hysteresis, which is typical for type IV isotherms and is indicative of capillary condensation taking place in mesopores. The TGA showed mass losses around 400 °C for 2-Pre and 2-Func, while 2-Ex decomposed at around 350 °C (Fig. S17†). The relative mass loss for 2-Func is *ca.* 4% lower than that for UiO-67-bpy, while the mass loss for 2-Ex is *ca.* 60% lower than that for UiO-67. The latter difference indicates missing linker defects in the structure of 2-Ex.

SEM (Fig. S21†) showed that the MOF crystallites have octahedral shapes, and that they are smaller for 2-Pre (0.2–0.5 μm) than for 2-Func (0.5–2 μm) and 2-Ex (0.5–1.5 μm). EDS analysis led to estimated Ru : Zr ratios of 0.08 (2-Pre), 0.05 (2-Func), and 0.18 (2-Ex). The values for 2-Pre and 2-Func are within expected values, just as discussed for the corresponding MOFs functionalized with complex 1 (see above). 2-Func was also synthesized using UiO-67 with 10% bpydc, which resulted in the same Ru : Zr ratio as for UiO-67 with 5% bpydc (the crystallites of both MOFs were of approximately the same sizes). This suggests that steric factors may contribute to limit the amount of incorporated Ru(ii) complex in the MOF. The high Ru : Zr ratio in 2-Ex are in agreement with both its dark purple color and its relatively low surface area (the Ru(ii) complexes should occupy available pore space in the MOF).

Digestion of the MOFs and subsequent ^1^H NMR analysis of the resulting solutions revealed that the signals belonging to complex 2 were apparent in the spectra of digested 2-Func (Fig. S34†) and 2-Ex (Fig. S35†). These signals were not visible in the spectra of digested 2-Pre (Fig. S33†), which could indicate that complex 2 is not incorporated at all, or that it decomposes during the MOF synthesis.

**Fig. 3:**
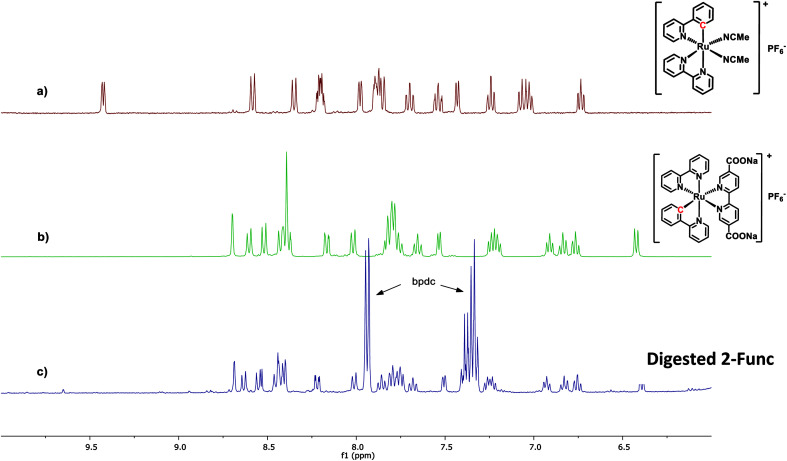
^1^H NMR spectra of (a) the Ru(ii) precursor *cis*-[Ru(ppy)(bpy)(MeCN)_2_]PF_6_, (b) complex 2, and (c) 2-Func digested in 1 M NaOH. All spectra are recorded in CD_3_OD.

In the case of 2-Func, this NMR result provides valuable information about the incorporation of complex 2, just as discussed above for 1-Func. In Fig. 3 the ^1^H NMR spectrum of digested 2-Func is shown together with the spectra of complex 2, which is the integrated product of the postsynthetic functionalization reaction, and of *cis*-[Ru(ppy)(bpy)(MeCN)_2_]PF_6_, which is the molecular Ru(ii) precursor for the reaction. The ^1^H NMR signals arising from complex 2 are clearly apparent in the spectrum of the digested MOF, while there is no indication of the reactant *cis*-[Ru(ppy)(bpy)(MeCN)_2_]PF_6_ (the extra signals result from the standard bpdc linkers). In the digestion process, the Zr-carboxylate bonds are analysis provides good evidence for the chemical incorporation of complex 2 into the MOF structure, as a consequence of the postsynthetic functionalization reaction between the bpydc linkers in the MOF and the complex *cis*-[Ru(ppy)(bpy)(MeCN)_2_]PF_6_.

The following observation relates to a particular mechanistic detail that highlights interesting similarities in mechanisms of Ru(ii) reactivity of molecular complexes in homogeneous solutions and during heterogeneous MOF construction processes. In the molecular precursor *cis*-[Ru(ppy)(bpy)(MeCN)_2_]PF_6_, the Ru-bonded C atom of the cyclometalated ppy ligand is located trans to a bpy N atom. When this species is converted to 2 by substitution of the two MeCN ligands with diethyl 2,2′-bipyridine-5,5′-dicarboxylate, the Ru-bonded C-atom of the ppy is instead found to occupy a position trans to the incoming (diethyl bpydc) ligand. This change in stereochemistry at Ru was demonstrated by ^1^H NMR as well as single-crystal X-ray diffraction analysis of the product 2.^36^ The isomer of 2, in which the C atom is trans to bpy, was found to have a significantly different ^1^H NMR spectrum than 2. It is interesting to see that during the coordination reaction (postsynthetic functionalization reaction) that occurs when UiO-67-bpy with 5% bpydc is reacted with *cis*-[Ru(ppy)(bpy)(MeCN)_2_]PF_6_, the same change in stereochemistry is observed at Ru.

**Fig. 4:**
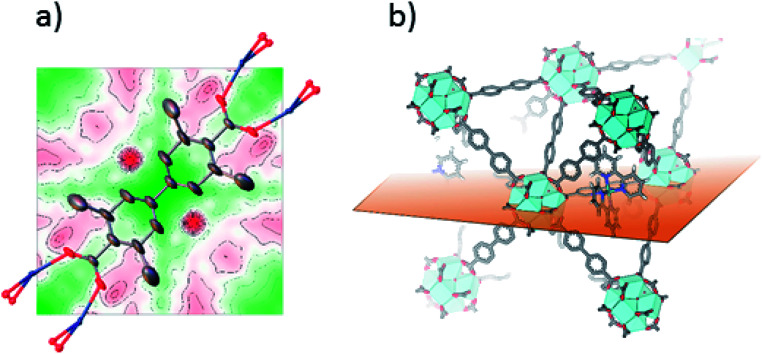
(a) 2D Fourier difference map of 2-Func, parallel to Miller plane 1 0 0, intersecting the expected position of Ru before including it in the crystal structure refinement. Ru is disordered over two symmetry equivalent positions on each side of the linker's axis of connectivity. (b) Representation of the plane used for the Fourier map in the structure of Ru-functionalized UiO-67-Me_2_-bpy.

SC-XRD was also performed on single crystals of 2-Func with 10% bpydc. As can be seen in Fig. 4a, the Fourier difference map for 2-Func clearly shows the presence of an atom in the expected position of Ru coordinated to a bipyridine linker. The occupancy coefficient of Ru refined freely to 8.4%. Just as for 1-Func, this is, in addition to the NMR result, direct evidence for the proper chemical incorporation of complex 2 as linker in UiO-67.

### Incorporation of complex 3

This Ru(ii) complex is similar to complex 2, except that the Ru-bonded C atom is located on the ligand with the carboxylate groups. Complex 3 was also shown to absorb intensely in the visible region, with corresponding MLCT transitions onto the carboxylated ligand.^36^ The complex was attempted incorporated into UiO-67 *via* premade linker synthesis (3-Pre) and postsynthetic linker exchange (3-Ex). Postsynthetic functionalization is not possible to employ for the incorporation of complex 3, since the complex is synthesized *via* base promoted C–H activation,^36^ which is not feasible for reaction with the MOF structure. The powder of 3-Pre was completely white. The same variations in synthesis conditions as for 2-Pre, and in addition, the use of 0.2 equivalents of Ru(ii) complex instead of 0.1 (relative to ZrCl_4_), was used, without any changes in the appearance of the powder. 3-Ex, on the other hand, had a dark maroon color. Both MOFs had the crystal structure of UiO-67 (Fig. S5†). The BET surface areas were measured to be 2541 and 1405 m^2^ g^−1^ for 3-Pre and 3-Ex, respectively. The isotherms show type II and type I behavior, respectively (Fig. S12 and S13†). TGA showed that the materials were stable up to *ca.* 480 (3-Pre) and 390 (3-Ex) °C (Fig. S18†). The relative mass loss for 3-Ex is slightly higher than that for pristine UiO-67, so there is probably not any significant amount of missing linker defects. SEM revealed octahedral crystallites with sizes in the ranges 0.2–0.5 (3-Pre) and 1–2 (3-Ex) μm (Fig. S22†). The Ru : Zr ratios obtained from EDS analysis were 0.01 for 3-Pre and 0.20 for 3-Ex. These values are in agreement with both the colors and the BET surface areas of the samples. The Ru : Zr ratio of 3-Ex is quite similar to that of 2-Ex (0.18), although their surface areas are significantly different (1405 *versus* 940 m^2^ g^−1^). In addition to its lower surface area, 2-Ex also shows poorer crystallinity and lower thermal stability. Thus, this could be a more defect MOF, which could explain the lower surface area.

^1^H NMR analysis of the digested MOF solutions showed that there were no signals from complex 3 in the spectra of digested 3-Pre (indicating no incorporation, Fig. S36†), while they were very clear in the spectra of 3-Ex (Fig. S37†).

### Incorporation of complex 4

**Fig. 5:**
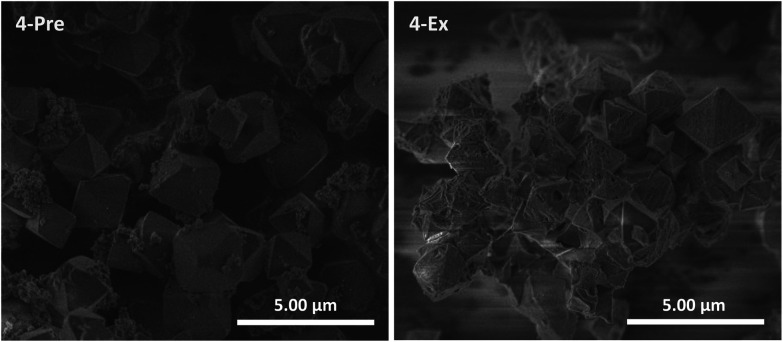
SEM images of the MOFs 4-Pre and 4-Ex.

**Fig. 6:**
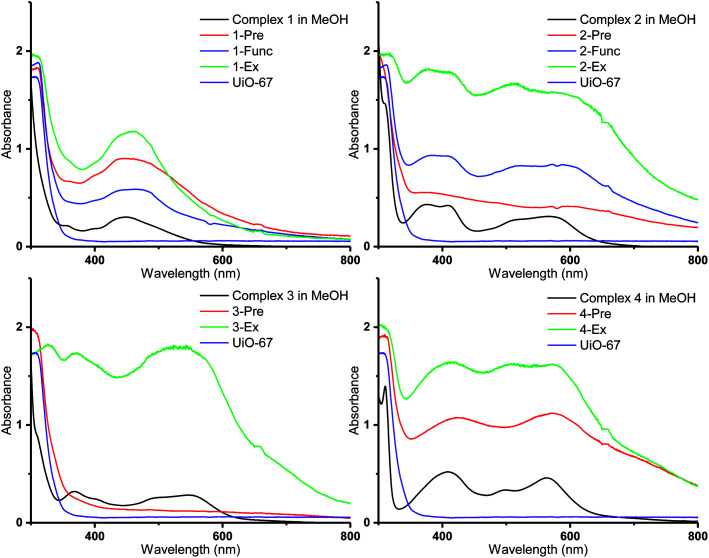
DR UV-Vis spectra of the Ru(ii)-functionalized MOFs compared to the solution spectra of the corresponding Ru(ii) complexes in methanol (black lines).

This cyclometalated Ru(ii) complex is known as a sensitizer in DSSCs.^29,31,37,38^ Unlike complexes 1–3, it does not contain a ligand with carboxylate groups oriented in a linear fashion, *i.e.* it is not strictly analogous, neither topologically nor in size, to the bpdc linker in UiO-67. Nevertheless, 4 was included in this work despite its geometry and attempted incorporated into UiO-67 using premade linker synthesis (4-Pre) and postsynthetic linker exchange (4-Ex). Both MOFs had a dark purple color. PXRD showed that both retained the crystal structure of UiO-67, although 4-Ex was slightly less crystalline than 4-Pre (Fig. S5†). The BET surface areas were measured to be 1530 and 761 m^2^ g^−1^ for 4-Pre and 4-Ex, respectively. 4-Pre has characteristics of a type II isotherm (Fig. S14†), while the isotherm of 4-Ex shows hysteresis typical for type IV and mesoporosity (Fig. S15†), similar to 2-Ex (Fig. S11†). TGA showed that the MOFs were stable up to *ca.* 400 (4-Pre) and 300 (4-Ex) °C. The relative mass loss for 4-Ex is *ca.* 100% lower than that for pristine UiO-67. This indicates a considerable amount of missing linker defects in 4-Ex. In the DSC signal of 4-Pre, there are two significant peaks. In Fig. S19,† this signal is compared to that of the molecular complex 4. The lowest temperature peak for 4-Pre coincides with the largest peak for complex 4. SEM revealed octahedral crystallites with sizes in the ranges 0.75–3 (4-Pre) and 0.5–2 (4-Ex) μm (Fig. 5). The crystallites of 4-Pre are covered with small agglomerates that could possibly be residual Ru(ii) complex that did not disappear during the washing procedure. These could be the phases that burn separately from the main MOF material, and give rise to the extra peak in the DSC signal. The surfaces of the crystallites of 4-Ex are somewhat more eroded than those of the other MOFs in this work. This is in agreement with the lower crystallinity, porosity, thermal stability, and relative mass loss of 4-Ex compared to the other MOFs, mentioned above. The Ru : Zr ratios obtained from EDS analysis were 0.16 for 4-Pre and 0.10 for 4-Ex. ^1^H NMR analysis of the digested MOF solutions showed that the signals from complex 4 are clearly apparent in the spectra of digested 4-Ex (Fig. S39†). They are, on the other hand, not clearly visible in the spectra of digested 4-Pre (Fig. S38†). This is surprising, given that 4-Pre has a UV-Vis absorption spectrum that is very similar to the spectrum of complex 4 in solution (Fig. 6, *vide infra*).

Based on the above results, it can be hypothesized that complex 4 can possibly be incorporated into the MOF by different manners. For example, one or more of its four carboxylate groups might connect at missing linker defects^59–63^ in the MOF structure. Alternatively, the complex may bind to the surface of the MOF crystallites, in a fashion similar to the bonding of sensitizers to TiO_2_ particles in DSSCs.^29,31,37,38^

### UV-visible spectroscopy

Diffuse reflectance UV-visible (DR UV-Vis) spectroscopy was employed in order to gain detailed information about the electronic properties of the MOFs. The absorbances of the MOFs are shown together with those of the corresponding molecular Ru(ii) complexes in methanol solution in Fig. 6 (3-Func and 4-Func are not available, as previously pointed out). It is apparent that the MOFs that are functionalized with cyclometalated Ru(ii) complexes (2–4) have absorption bands that extend significantly further into the visible region, compared to the MOFs that are functionalized with complex 1. Qualitatively (with two exceptions), the main features of the absorption profiles of the molecular complexes 1–4 are apparent in the spectra of all the MOFs functionalized with the respective Ru(ii) moieties. This indicates that the complexes retain their integrity during the functionalization reactions. The two exceptions are 2-Pre, which shows a relatively flat absorption profile over the entire visible region, and 3-Pre, which absorbs rather weakly in the same region. This is in qualitative agreement with their grey and white appearances, respectively.

As shown in our previous article, using TD-DFT (time dependent density functional theory) calculations, the absorption bands of Ru(ii) complexes 1–4 in the visible region are due to MLCT (metal–ligand charge transfer) transitions, in which some of the most intense populate the carboxylated ligands.^36^ When the complexes are incorporated into MOFs, these transitions could lead to electron transfer into the MOF structures *via* the carboxylate groups, as proposed for other photosensitizer-MOF systems in previous reports.^32,33,64–69^ This would be beneficial if the MOFs were to be used as photocatalysts for reactions such as photocatalytic CO_2_ reduction.

Interestingly, the resemblance between the absorption profiles of 2-Func and complex 2 indicates that the stereochemistry of the incorporated Ru(ii) complex is the same as for complex 2 (ppy-C *trans* to bpydc), thus different from the precursor *cis*-[Ru(ppy)(bpy)(MeCN)_2_]PF_6_ (ppy-C *trans* to bpy), as already discussed for the NMR results, and in our previous work.^36^

The MOFs functionalized by postsynthetic linker exchange have the highest absorption intensities, compared to those prepared by the other methods. This could be due to a higher portion of the Ru(ii) complexes being located on, or close to, the surface of the crystallites as a result of this functionalization method. Indeed, EDS showed that 2-Ex and 3-Ex have higher Ru : Zr ratios compared to the corresponding MOFs functionalized by premade linker synthesis and postsynthetic functionalization.

## Conclusions

In this work, we have successfully incorporated four different Ru(ii) complexes into the MOF UiO-67 using three different synthetic strategies: premade linker synthesis, postsynthetic functionalization, and postsynthetic linker exchange. Three of these Ru(ii) complexes are cyclometalated (complexes 2, 3 and 4), which gives the corresponding Ru(ii)-functionalized MOFs an increased absorbance in the visible region, compared to those functionalized with the non-cyclometalated reference (complex 1). Such broadband absorption is important since it opens for a much better use of direct sunlight for various applications. Combined with their high porosities and thermal stabilities, this property makes these Ru(ii)-functionalized MOFs promising as efficient heterogeneous photocatalysts.

## Author contributions

The manuscript was written through contributions of all authors. All authors have given approval to the final version of the manuscript.

## Conflicts of interest

There are no conflicts to declare.

## Supplementary Material

RA-010-C9RA06984D-s001

RA-010-C9RA06984D-s002
