# Supplementary material for: Strongly visible light-absorbing metal–organic frameworks functionalized by cyclometalated ruthenium(ii) complexes
Source: RSC Adv. 2020 Mar 2;10(15):9052–62. doi: 10.1039/c9ra06984d (PMC9050028; doi:10.1039/c9ra06984d)
Supplement: RA-010-C9RA06984D-s001 [file RA-010-C9RA06984D-s001.pdf]

Electronic Supplementary Material (ESI) for RSC Advances.
This journal is © The Royal Society of Chemistry 2020

Supporting information for

Strongly Visible Light-Absorbing Metal-Organic Frameworks Functionalized by Cyclometalated Ruthenium(II) Complexes

*Eirik Mydske Thoresen, Sigurd Øien-Ødegaard, Gurpreet Kaur, Mats Tilset, Karl Petter Lillerud, and Mohamed Amedjkouh**

**E-mail: mamou@kjemi.uio.no.*

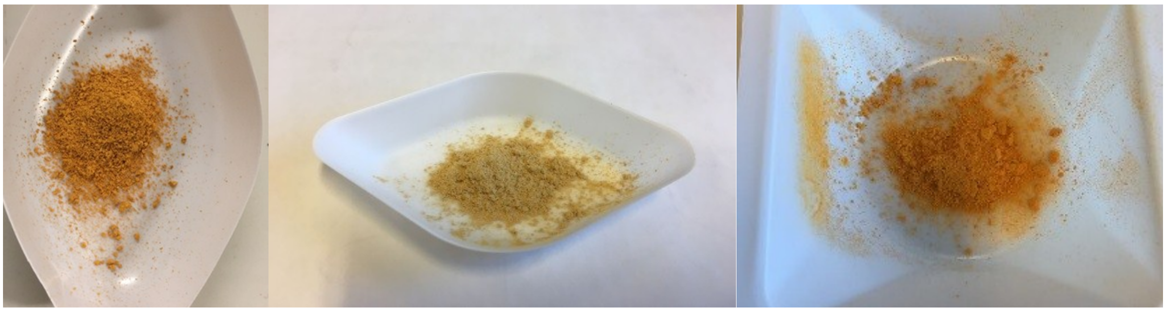

Figure S1. Pictures of the powders of (from left to right) 1-Pre, 1-Func, 1-Ex.

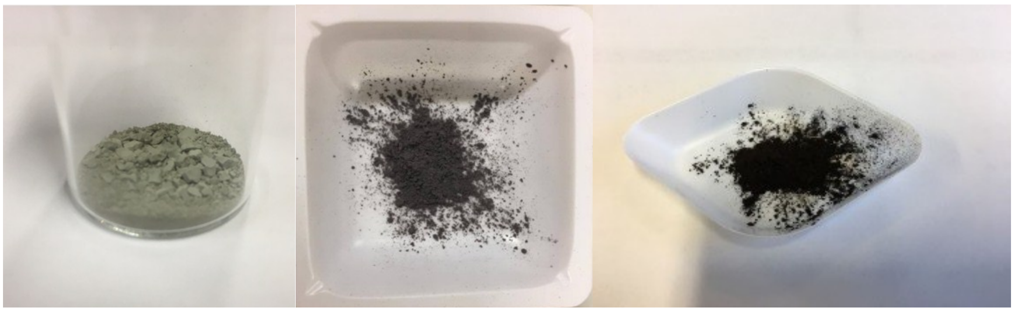

Figure S2. Pictures of the powders of (from left to right) 2-Pre, 2-Func, 2-Ex.

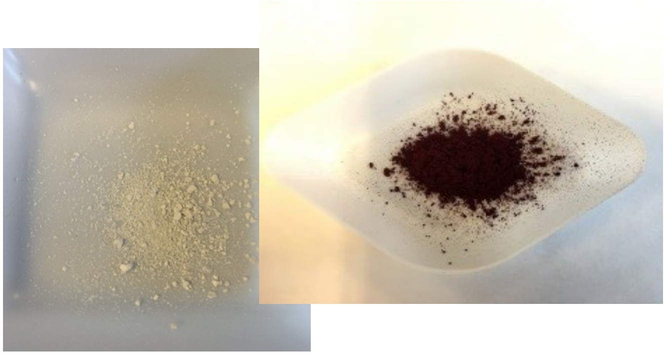

Figure S3. Pictures of the powders of (from left to right) 3-Pre, 3-Ex.

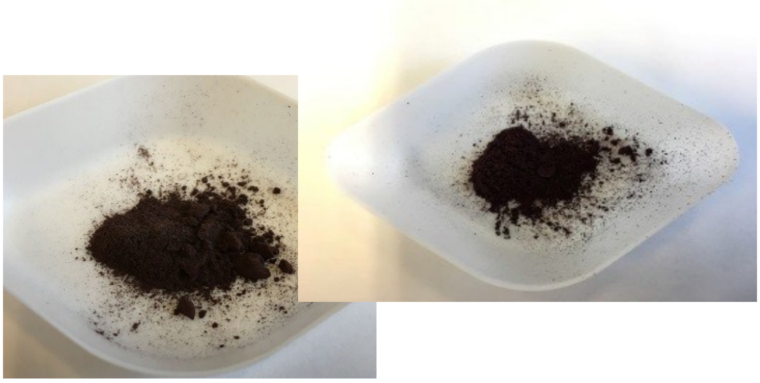

Figure S4. Pictures of the powders of (from left to right) 4-Pre, 4-Ex.

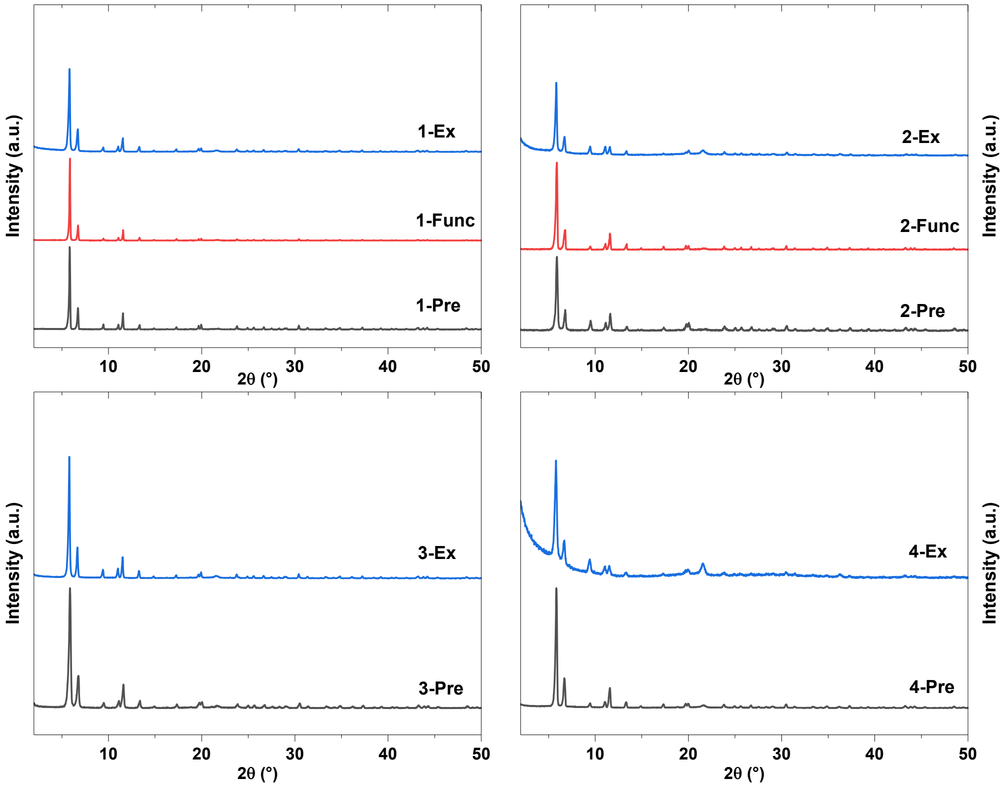

Figure S5. Powder X-ray diffraction patterns for the Ru(II)-functionalized UiO-67 MOFs.

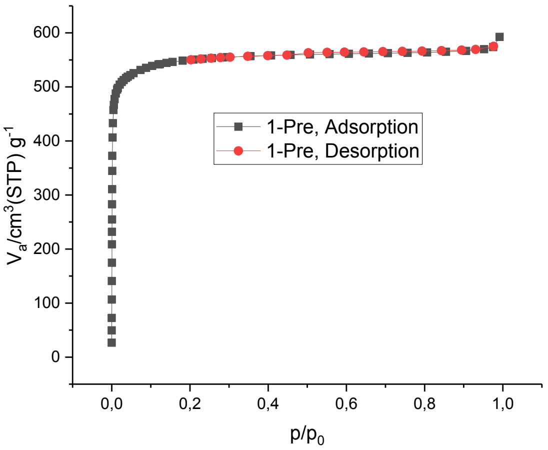

Figure S6. Adsorption/desorption isotherm for 1-Pre.

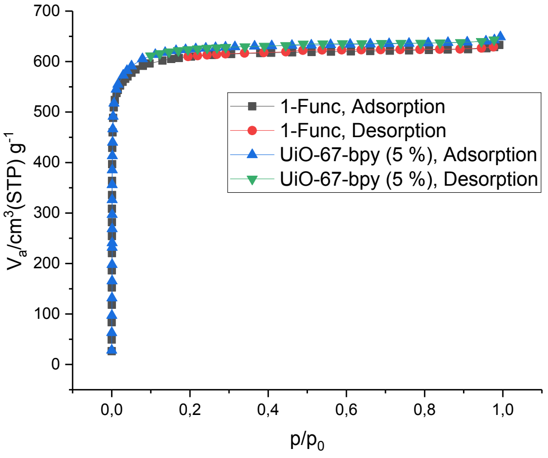

Figure S7. Adsorption/desorption isotherm for 1-Func and UiO-67-bpy (5 %).

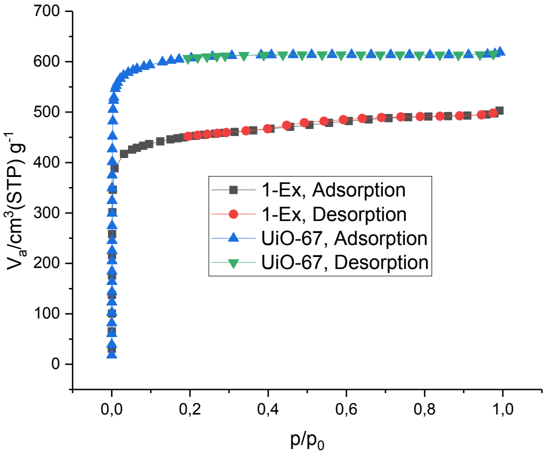

Figure S8. Adsorption/desorption isotherms for 1-Ex and UiO-67.

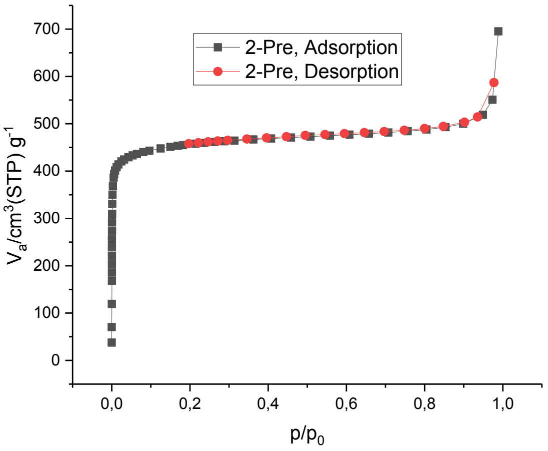

Figure S9. Adsorption/desorption isotherm for 2-Pre.

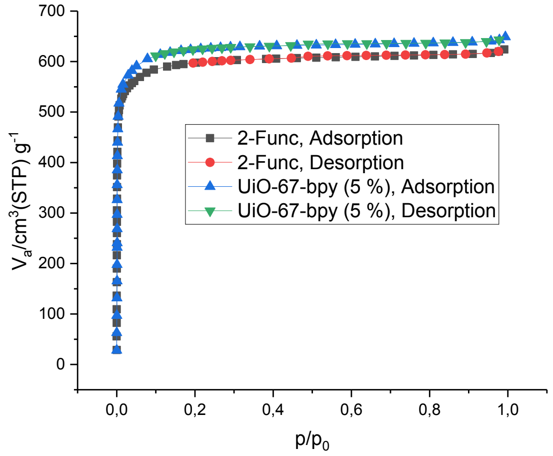

Figure S10. Adsorption/desorption isotherm for 2-Func and UiO-67-bpy (5 %).

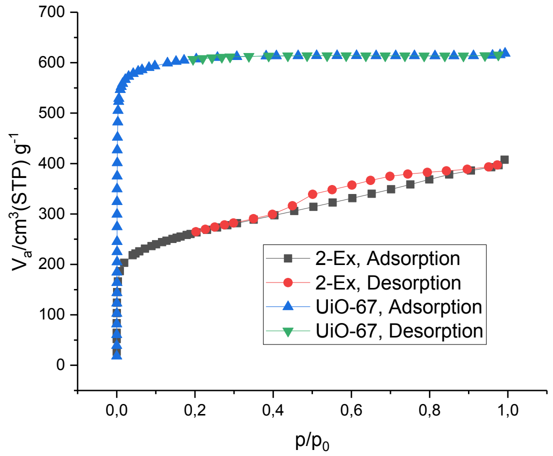

Figure S11. Adsorption/desorption isotherms for 2-Ex and UiO-67.

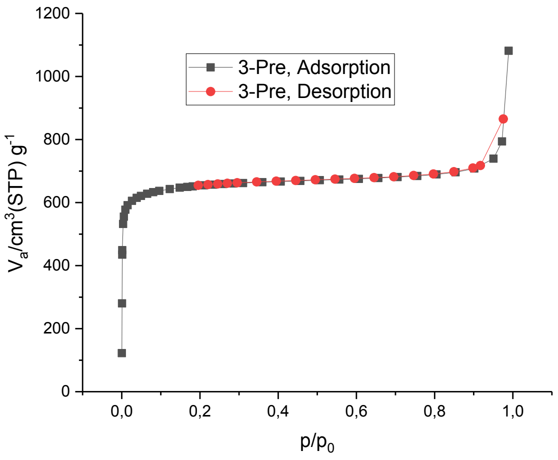

Figure S12. Adsorption/desorption isotherm for 3-Pre.

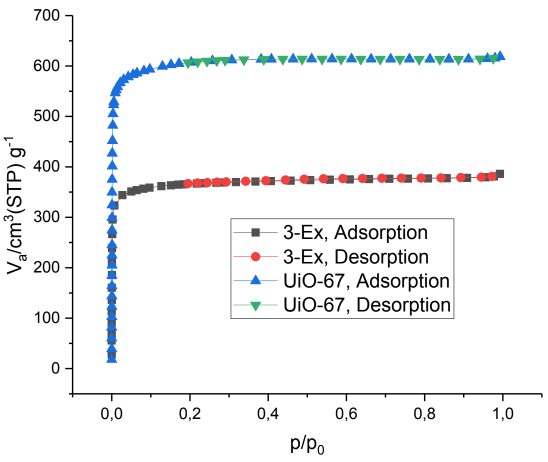

Figure S13. Adsorption/desorption isotherms for 3-Ex and UiO-67.

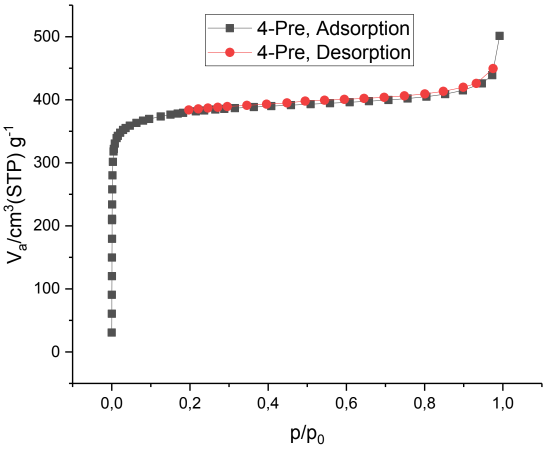

Figure S14. Adsorption/desorption isotherm for 4-Pre.

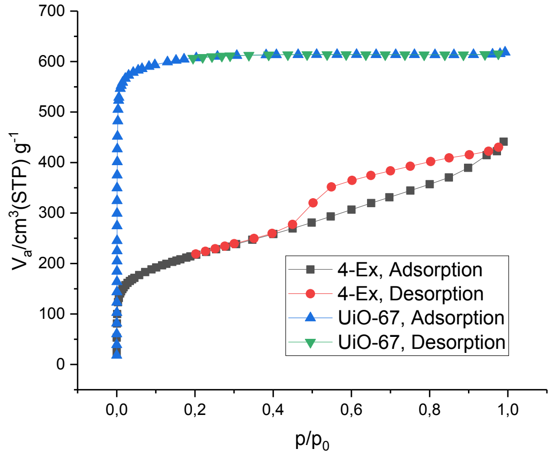

Figure S15. Adsorption/desorption isotherms for 4-Ex and UiO-67.

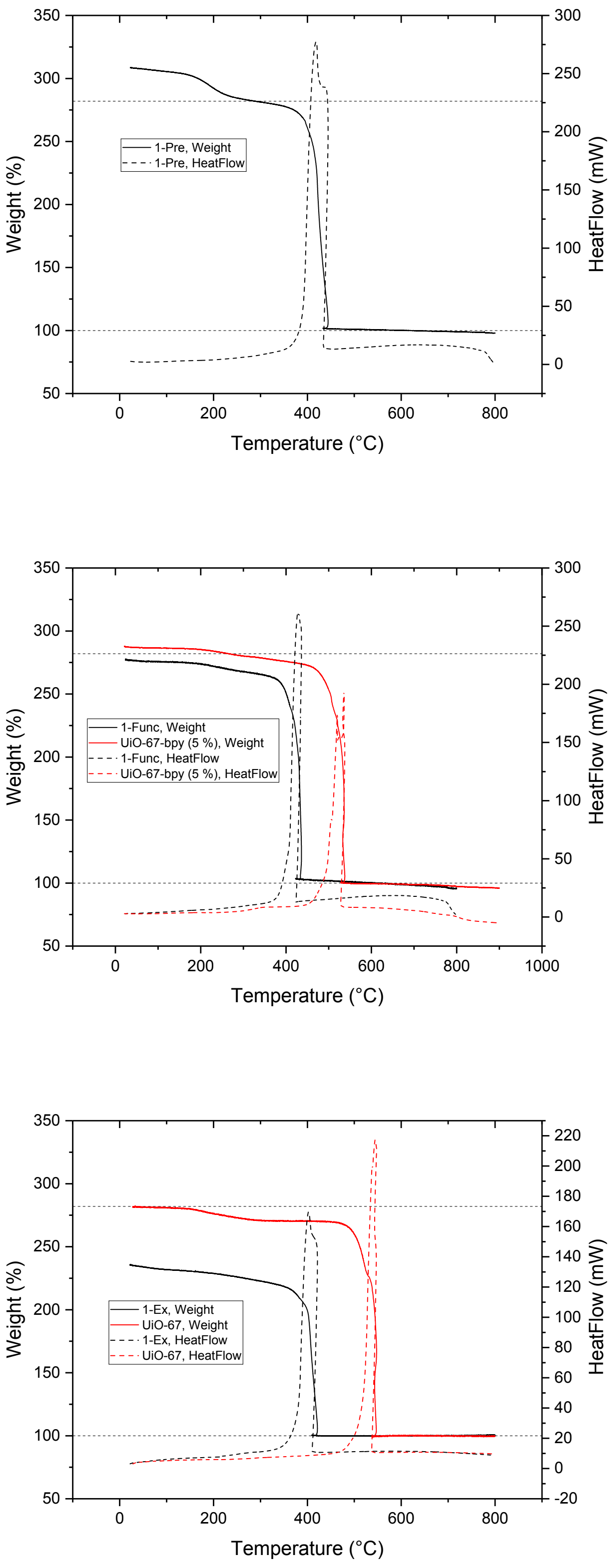

Figure S16. TGA-DSC of 1-Pre (top), 1-Func and UiO-67-bpy (5 %) (middle), and 1-Ex and UiO-67 (bottom). Solid curves, left axes – TGA traces (normalized such that end weights = 100 %). Dashed curves, right axes – DSC signals. The theoretical weight of ideal dehydroxylated UiO-67 are emphasized by the upper dashed, horizontal lines (282 %).

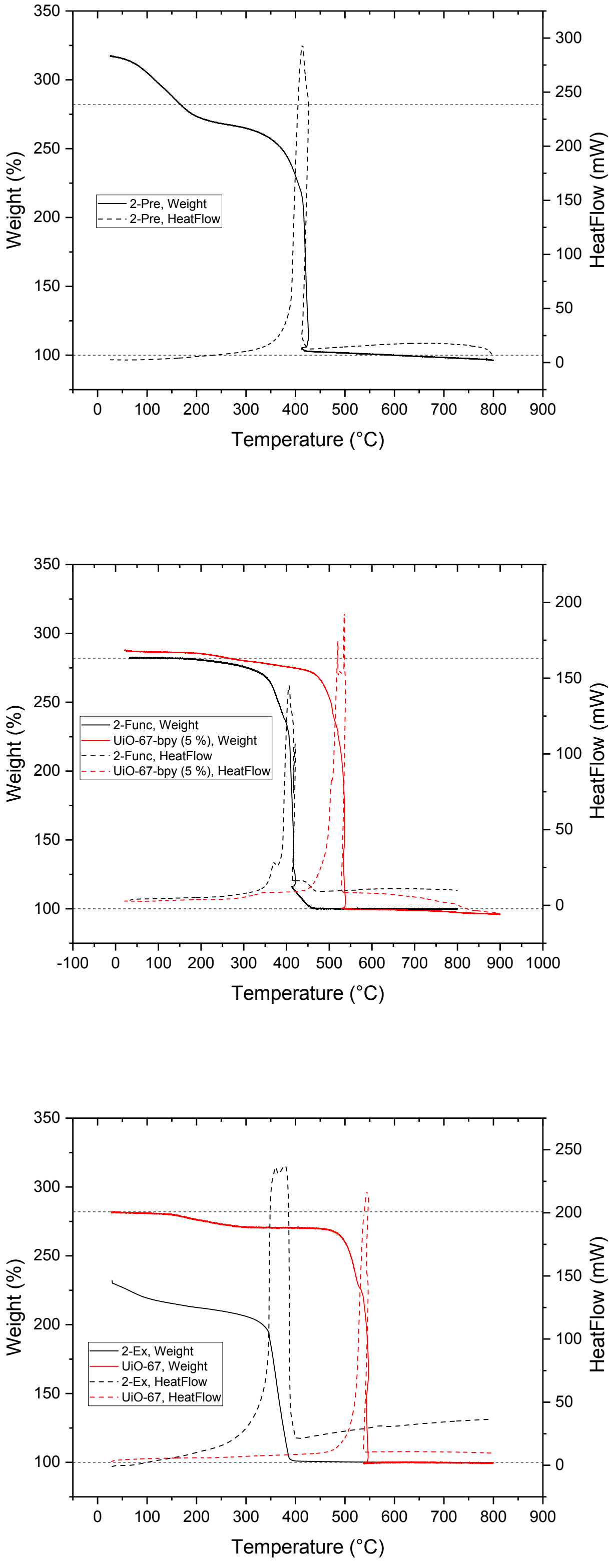

Figure S17. TGA-DSC of 2-Pre (top), 2-Func and UiO-67-bpy (5 %) (middle), and 2-Ex and UiO-67 (bottom). Solid curves, left axes – TGA traces (normalized such that end weights = 100 %). Dashed curves, right axes – DSC signals. The theoretical weight of ideal dehydroxylated UiO-67 are emphasized by the upper dashed, horizontal lines (282 %).

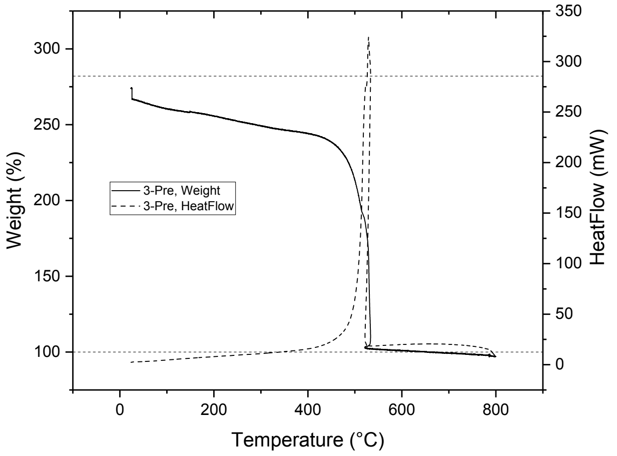

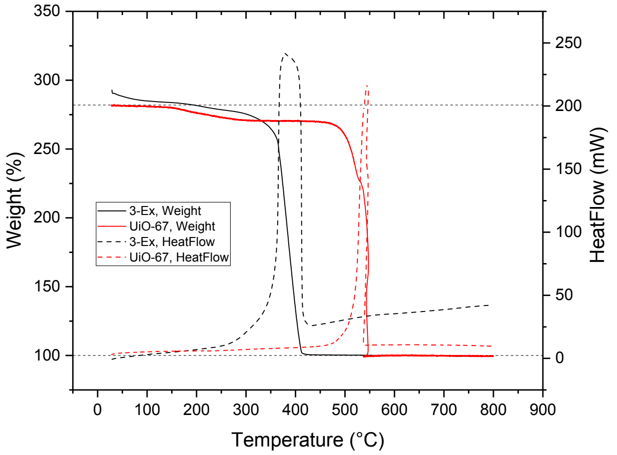

Figure S18. TGA-DSC of 3-Pre (top), and 3-Ex and UiO-67 (bottom). Solid curves, left axes – TGA traces (normalized such that end weights = 100 %). Dashed curves, right axes – DSC signals. The theoretical weight of ideal dehydroxylated UiO-67 are emphasized by the upper dashed, horizontal lines (282 %).

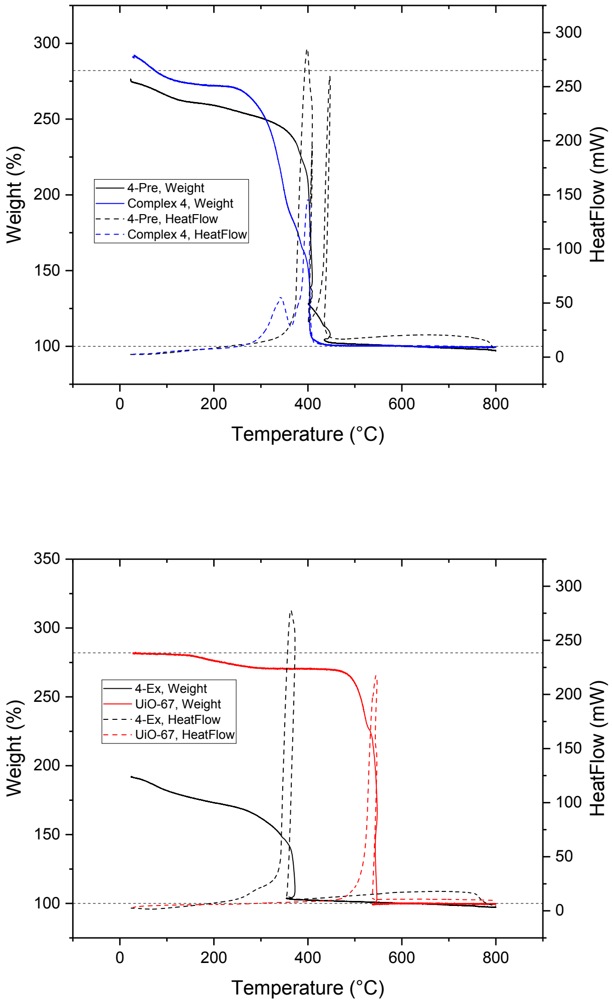

Figure S19. TGA-DSC of 4-Pre and complex 4 (top), and 4-Ex and UiO-67 (bottom). Solid curves, left axes – TGA traces (normalized such that end weights = 100 %). Dashed curves, right axes – DSC signals. The theoretical weight of ideal dehydroxylated UiO-67 are emphasized by the upper dashed, horizontal lines (282 %).

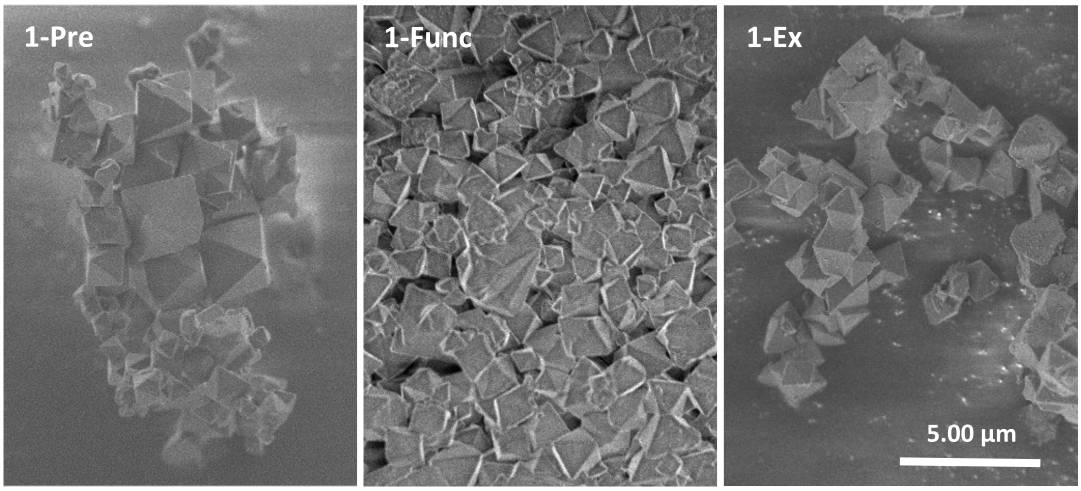

Figure S20. SEM images of the MOFs 1-Pre, 1-Func, and 1-Ex.

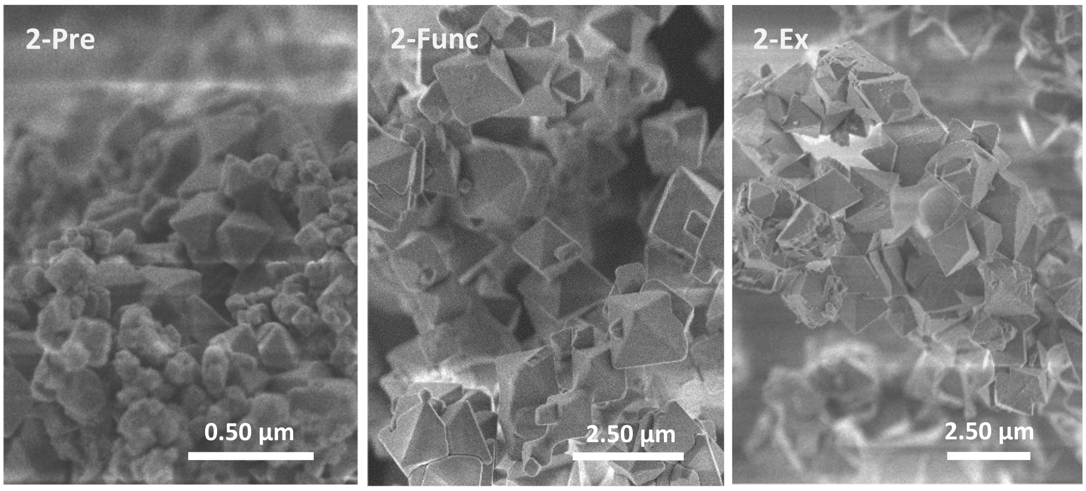

Figure S21. SEM images of the MOFs 2-Pre, 2-Func, and 2-Ex.

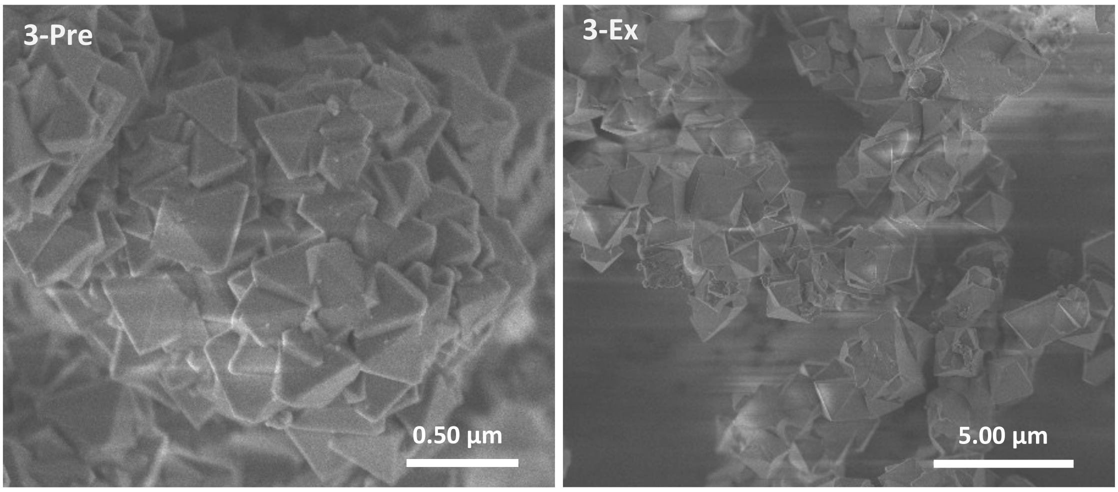

Figure S22. SEM images of the MOFs 3-Pre and 3-Ex.

c:\edax32\genesis\genspc.spc

Label:Chlorite (Nrm.%= 38.86, 20.96, 34.83, 1.14, 3.84, 0.28)

kV:10.0 Tilt:0.0 Take-off:35.3 Det Type:SUTW+ Res:130 Amp.T:102.4

FS : 11146 Lsec : 200 12-Mar-2019 19:02:11

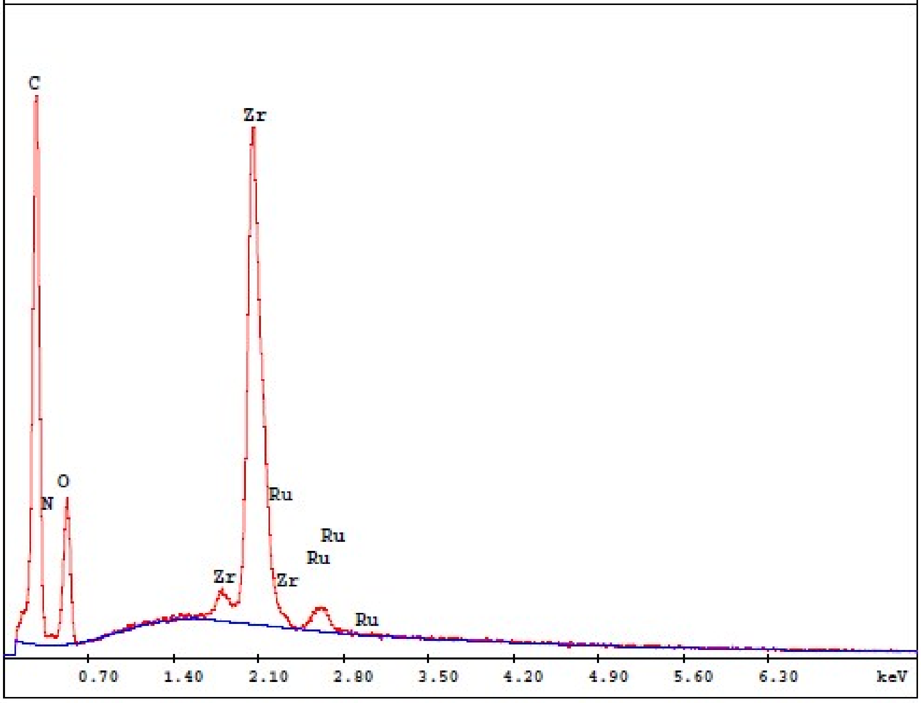

Element	Wt %	At %	K-Ratio	Z	A	F
C K	48.35	79.55	0.1664	1.1186	0.3077	1.0001
N K	1.51	2.13	0.0031	1.1052	0.1847	1.0001
O K	7.38	9.12	0.0232	1.0929	0.2876	1.0001
ZrL	39.85	8.63	0.3326	0.8037	1.0378	1.0007
RuL	2.91	0.57	0.0212	0.7863	0.9279	1.0000
Total	100.00	100.00				

Element	Net Inte.	Bkgd Inte.	Inte. Error	P/B
C K	237.57	4.91	0.47	48.43
N K	4.63	4.57	5.67	1.01
O K	62.35	4.93	0.96	12.66
ZrL	321.90	23.21	0.42	13.87
RuL	15.08	20.05	3.48	0.75

Figure S23. EDS spectrum and elemental analysis of 1-Pre.

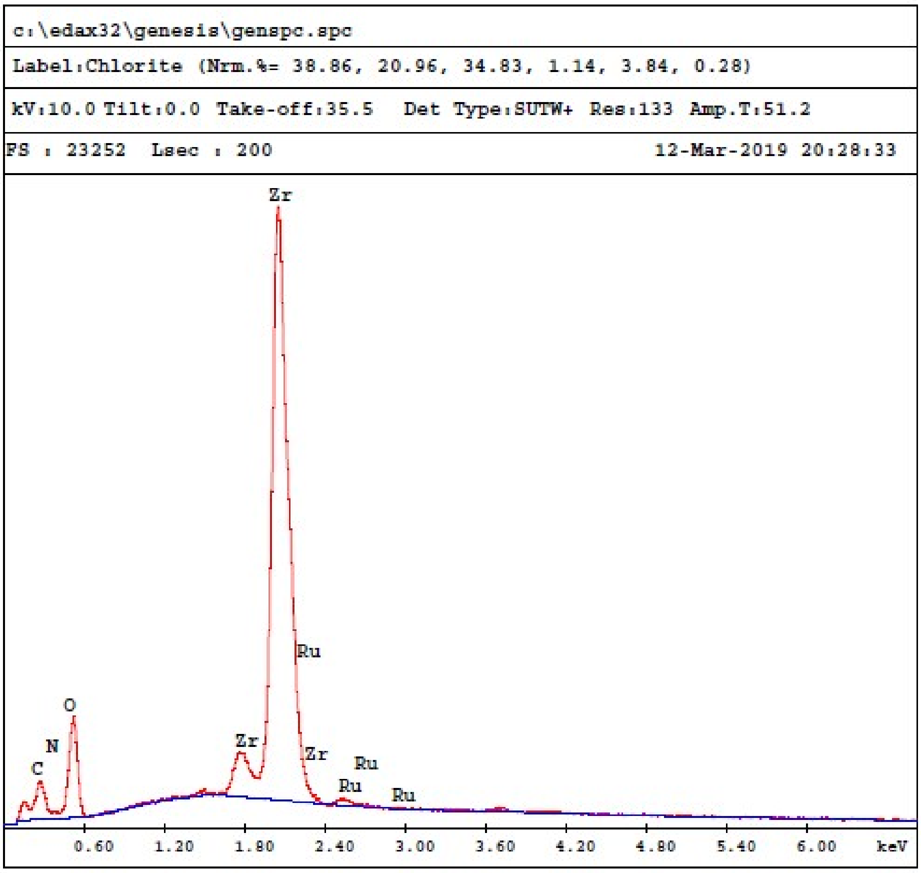

Element	Wt %	At %	K-Ratio	Z	A	F
C K	9.36	32.15	0.0209	1.2663	0.1765	1.0001
N K	1.78	5.23	0.0044	1.2505	0.1965	1.0002
O K	10.58	27.28	0.0349	1.2357	0.2666	1.0002
ZrL	76.74	34.71	0.7076	0.9123	1.0105	1.0002
RuL	1.55	0.63	0.0114	0.8945	0.8189	1.0000
Total	100.00	100.00				

Element	Net Inte.	Bkgd Inte.	Inte. Error	P/B
C K	35.74	4.68	1.33	7.64
N K	7.86	6.14	4.04	1.28
O K	112.01	8.70	0.72	12.87
ZrL	818.81	38.80	0.26	21.10
RuL	9.64	32.94	6.37	0.29

Figure S24. EDS spectrum and elemental analysis of 1-Ex.

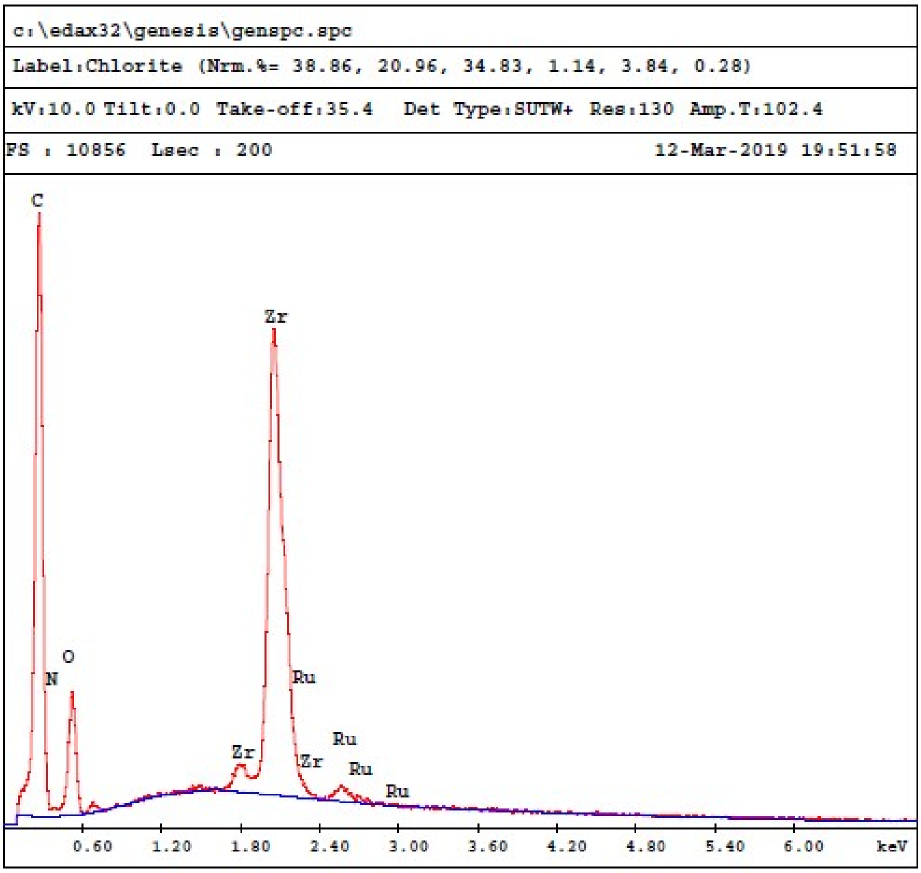

Element	Wt %	At %	K-Ratio	Z	A	F
C K	51.95	82.26	0.1841	1.1102	0.3192	1.0000
N K	1.29	1.75	0.0026	1.0970	0.1826	1.0001
O K	6.41	7.62	0.0201	1.0848	0.2888	1.0001
ZrL	38.27	7.98	0.3176	0.7974	1.0403	1.0005
RuL	2.08	0.39	0.0152	0.7801	0.9339	1.0000
Total	100.00	100.00				

Element	Net Inte.	Bkgd Inte.	Inte. Error	P/B
C K	250.01	3.80	0.45	65.79
N K	3.69	3.80	6.43	0.97
O K	51.29	4.32	1.07	11.87
ZrL	292.27	21.82	0.44	13.39
RuL	10.24	18.90	4.79	0.54

Figure S25. EDS spectrum and elemental analysis of 2-Func.

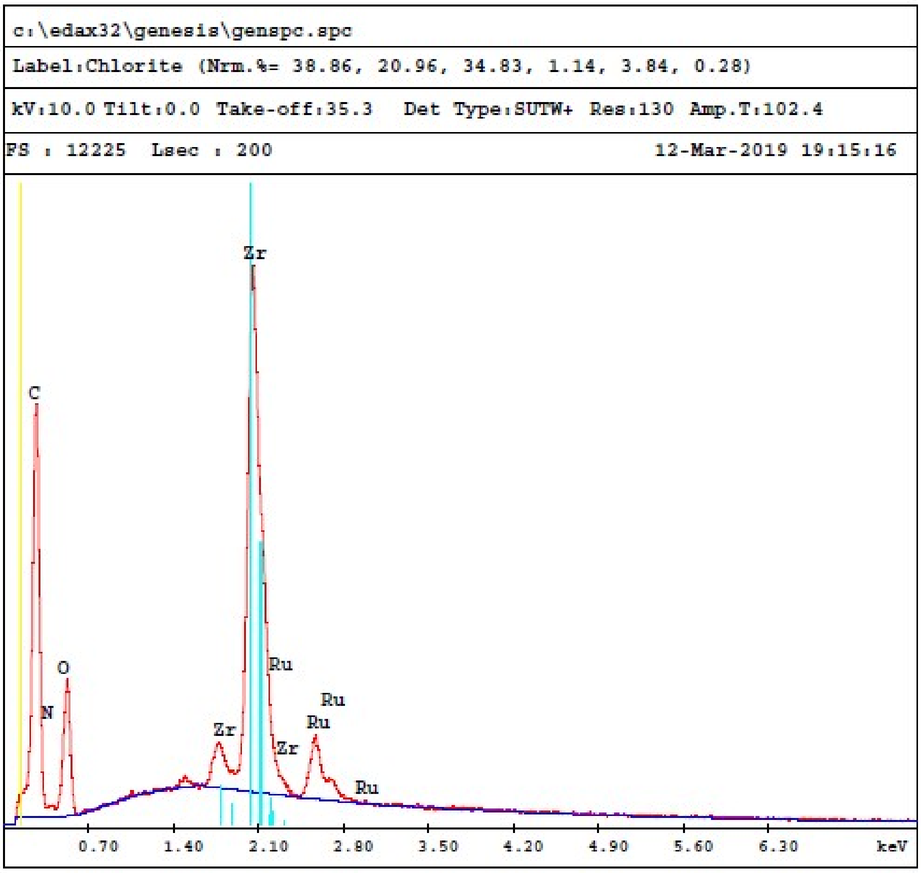

Element	Wt %	At %	K-Ratio	Z	A	F
C K	39.66	74.25	0.1320	1.1484	0.2897	1.0001
N K	1.93	3.09	0.0041	1.1345	0.1864	1.0001
O K	7.30	10.26	0.0227	1.1217	0.2777	1.0001
ZrL	42.77	10.54	0.3643	0.8258	1.0295	1.0017
RuL	8.33	1.85	0.0617	0.8084	0.9157	1.0000
Total	100.00	100.00				

Element	Net Inte.	Bkgd Inte.	Inte. Error	P/B
C K	195.10	3.65	0.52	53.38
N K	6.35	3.77	4.15	1.68
O K	63.30	4.39	0.95	14.42
ZrL	365.17	27.48	0.40	13.29
RuL	45.38	23.81	1.50	1.91

Figure S26. EDS spectrum and elemental analysis of 2-Ex.

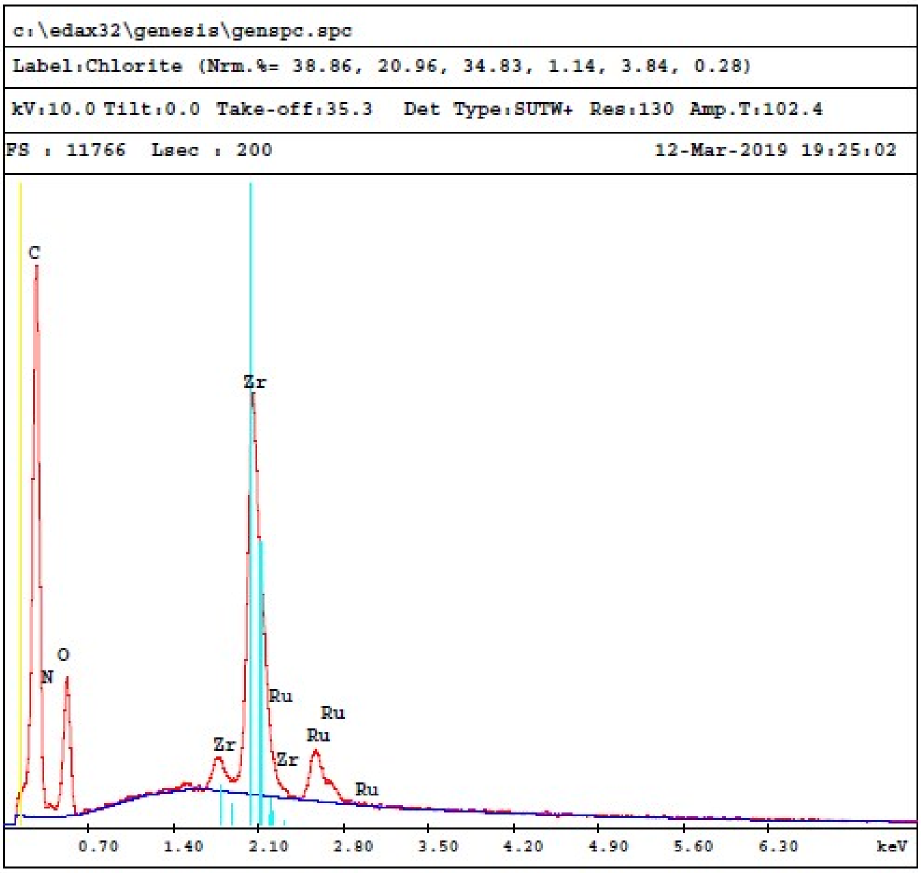

Element	Wt %	At %	K-Ratio	Z	A	F
C K	48.08	78.83	0.1819	1.1176	0.3386	1.0001
N K	1.97	2.77	0.0040	1.1042	0.1856	1.0001
O K	7.66	9.42	0.0239	1.0919	0.2855	1.0001
ZrL	34.75	7.50	0.2896	0.8030	1.0358	1.0018
RuL	7.54	1.47	0.0558	0.7857	0.9412	1.0000
Total	100.00	100.00				

Element	Net Inte.	Bkgd Inte.	Inte. Error	P/B
C K	249.12	3.86	0.45	64.62
N K	5.83	3.77	4.43	1.55
O K	61.51	4.22	0.96	14.59
ZrL	268.83	24.25	0.47	11.09
RuL	37.99	20.92	1.66	1.82

Figure S27. EDS spectrum and elemental analysis of 3-Ex.

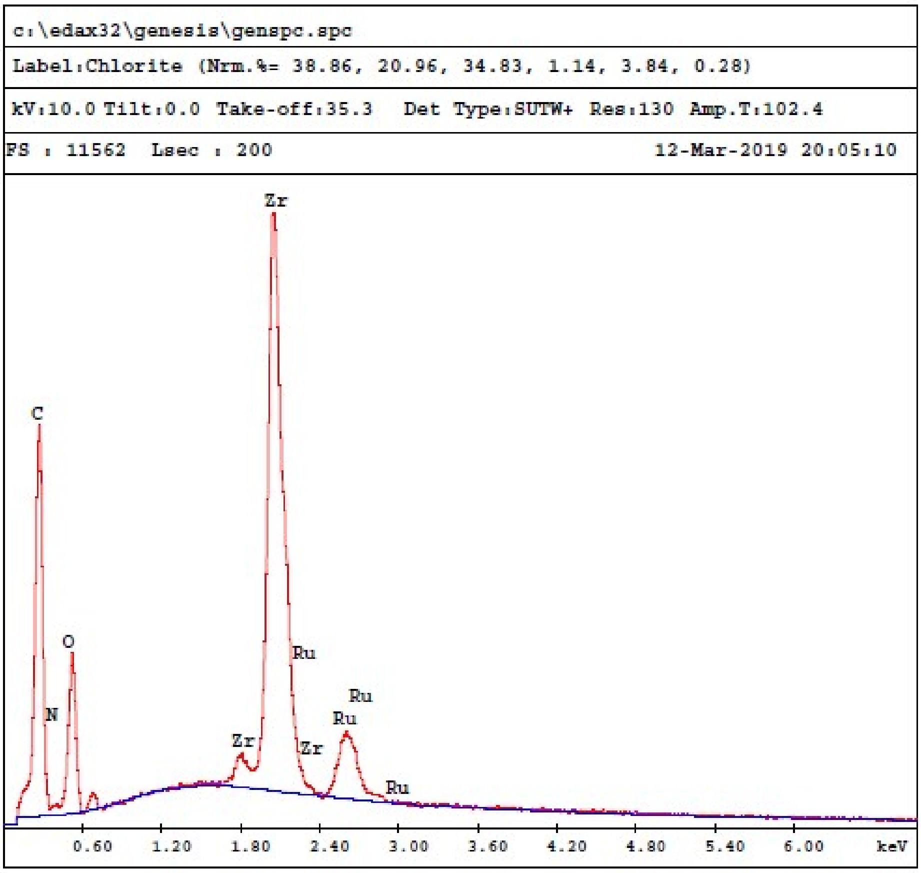

Element	Wt %	At %	K-Ratio	Z	A	F
C K	36.80	72.02	0.1169	1.1580	0.2743	1.0001
N K	1.54	2.59	0.0033	1.1440	0.1873	1.0001
O K	8.01	11.77	0.0252	1.1310	0.2777	1.0001
ZrL	45.77	11.79	0.3923	0.8329	1.0276	1.0016
RuL	7.88	1.83	0.0582	0.8155	0.9065	1.0000
Total	100.00	100.00				

Element	Net Inte.	Bkgd Inte.	Inte. Error	P/B
C K	172.45	4.14	0.55	41.65
N K	5.14	4.53	5.19	1.13
O K	69.88	5.49	0.91	12.74
ZrL	392.46	26.80	0.38	14.65
RuL	42.75	23.08	1.56	1.85

Figure S28. EDS spectrum and elemental analysis of 4-Pre.

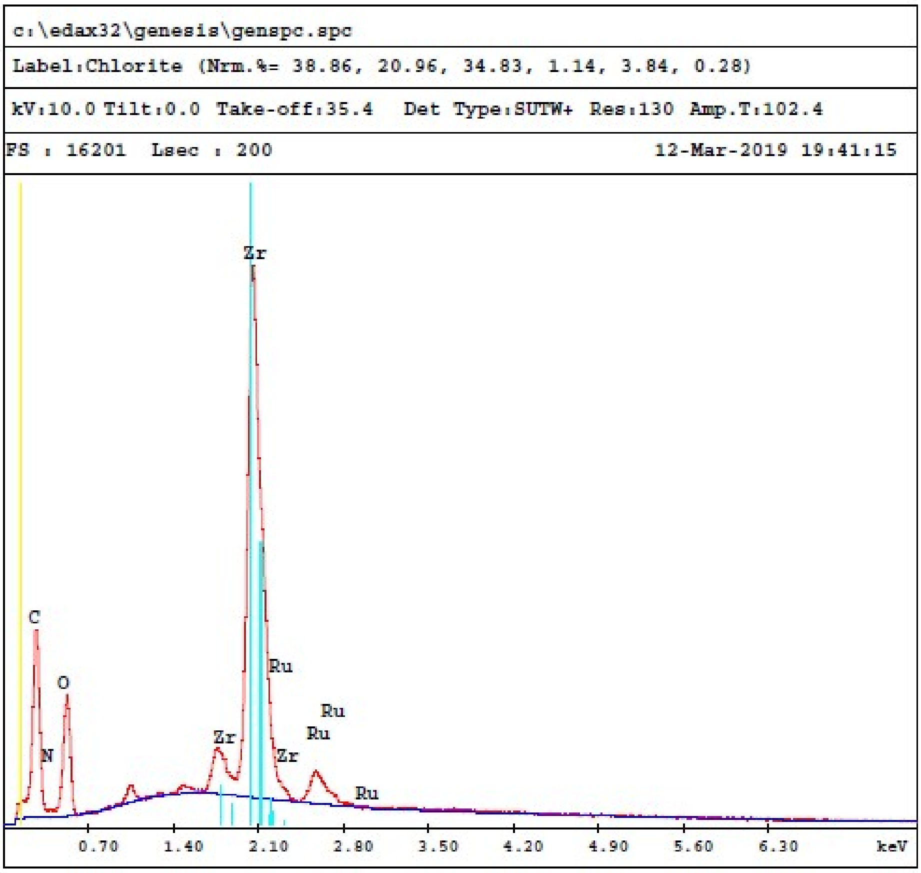

Element	Wt %	At %	K-Ratio	Z	A	F
C K	28.37	64.32	0.0791	1.1893	0.2342	1.0001
N K	1.59	3.09	0.0035	1.1748	0.1900	1.0001
O K	8.45	14.38	0.0269	1.1613	0.2741	1.0001
ZrL	55.66	16.61	0.4876	0.8560	1.0224	1.0011
RuL	5.93	1.60	0.0436	0.8385	0.8779	1.0000
Total	100.00	100.00				

Element	Net Inte.	Bkgd Inte.	Inte. Error	P/B
C K	118.58	4.41	0.67	26.89
N K	5.61	4.76	4.90	1.18
O K	75.92	5.70	0.87	13.33
ZrL	495.60	30.05	0.34	16.50
RuL	32.53	25.67	1.99	1.27

Figure S29. EDS spectrum and elemental analysis of 4-Ex.

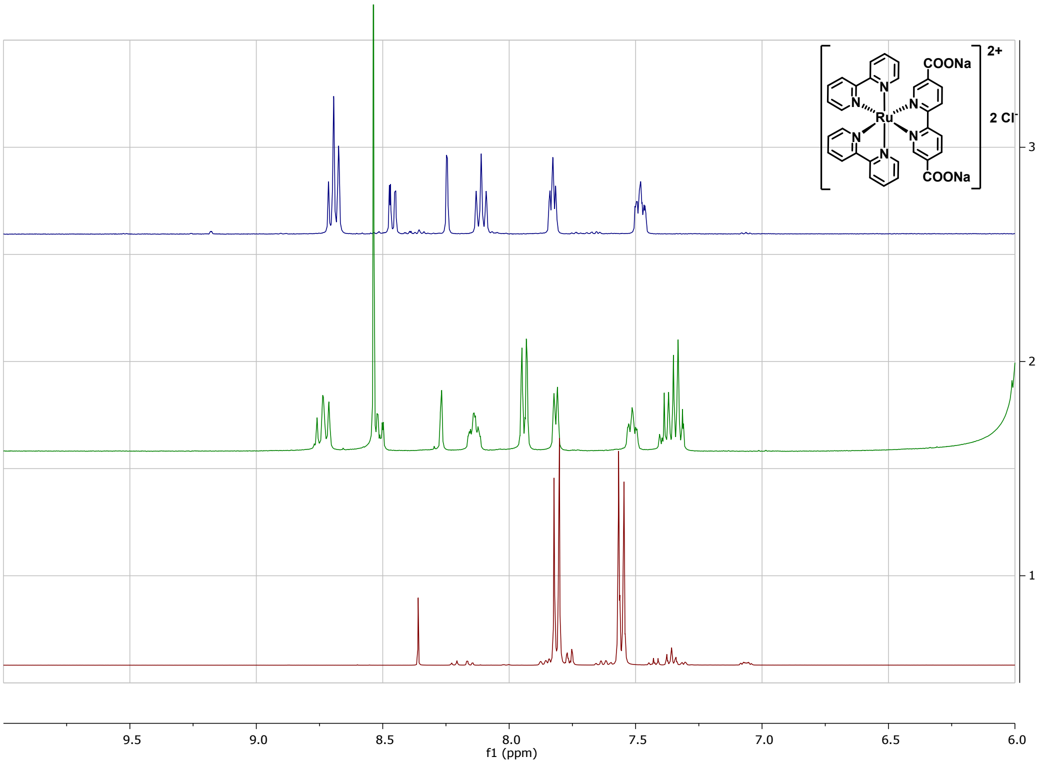

Figure S30. ^{1}H NMR spectra of 1-Pre digested in 1 M NaOH in D_2O (bottom), redissolved in CD_3OD (middle) and complex 1 in CD_3OD with a drop of 1 M NaOH in D_2O (top).

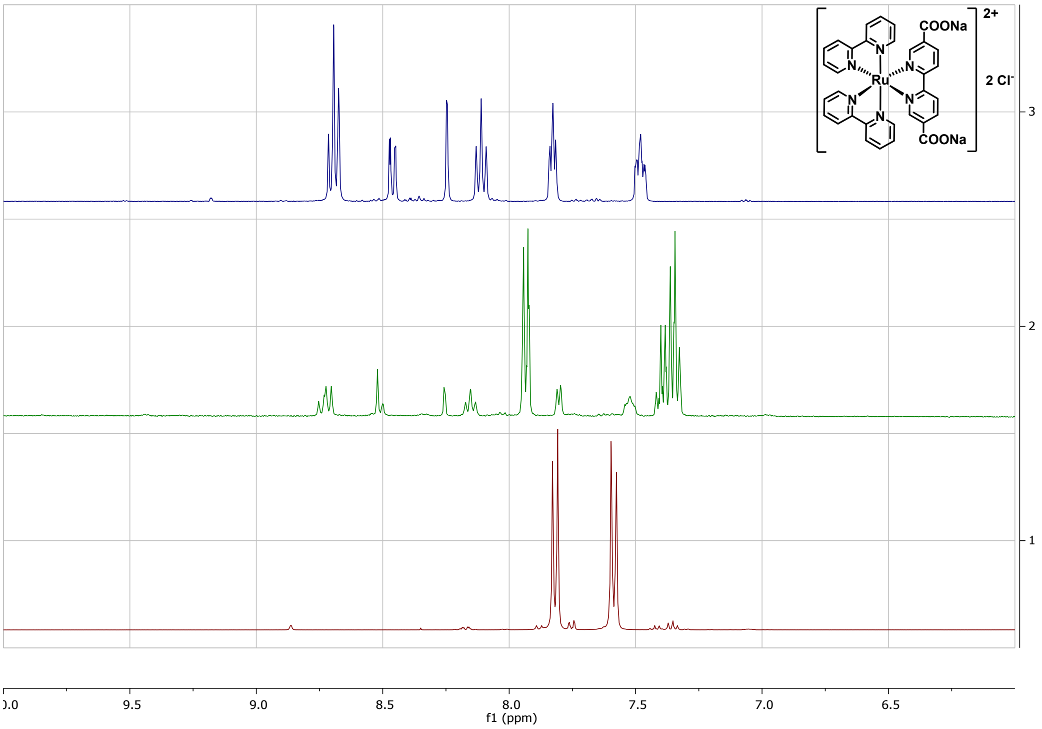

Figure S31. ^{1}H NMR spectra of 1-Func digested in 1 M NaOH in D_2O (bottom), redissolved in CD_3OD (middle) and complex 1 in CD_3OD with a drop of 1 M NaOH in D_2O (top).

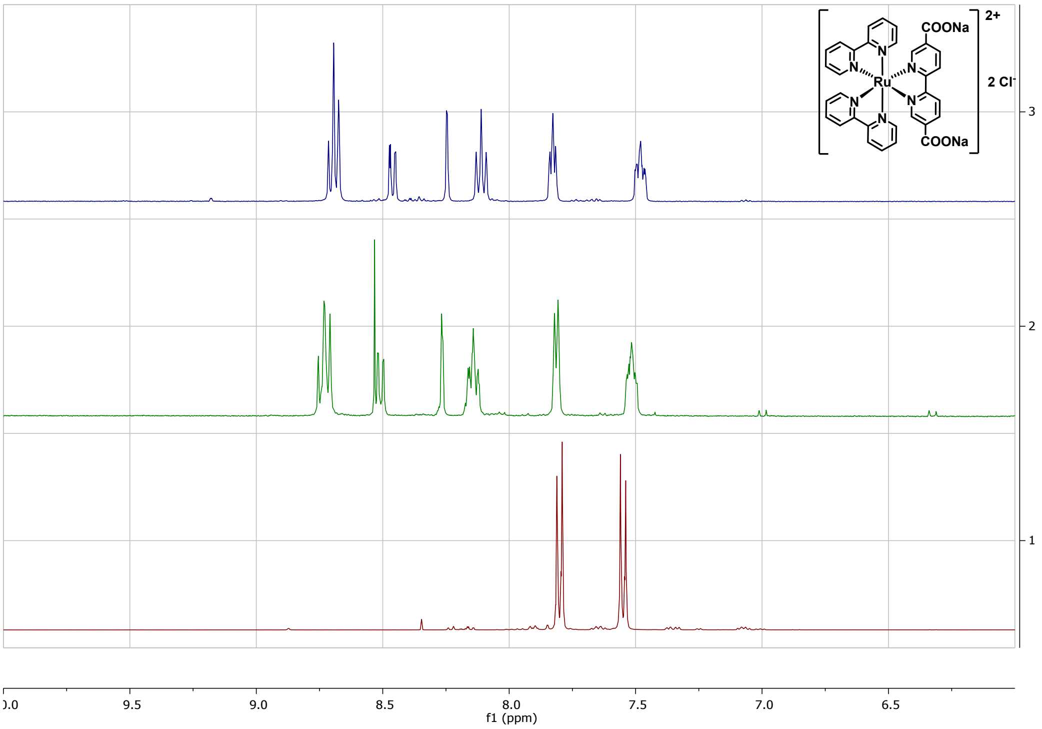

Figure S32. ^{1}**H NMR spectra of 1-Ex digested in 1 M NaOH in D_2O (bottom), redissolved in CD_3OD (middle) and complex 1 in CD_3OD with a drop of 1 M NaOH in D_2O (top).**

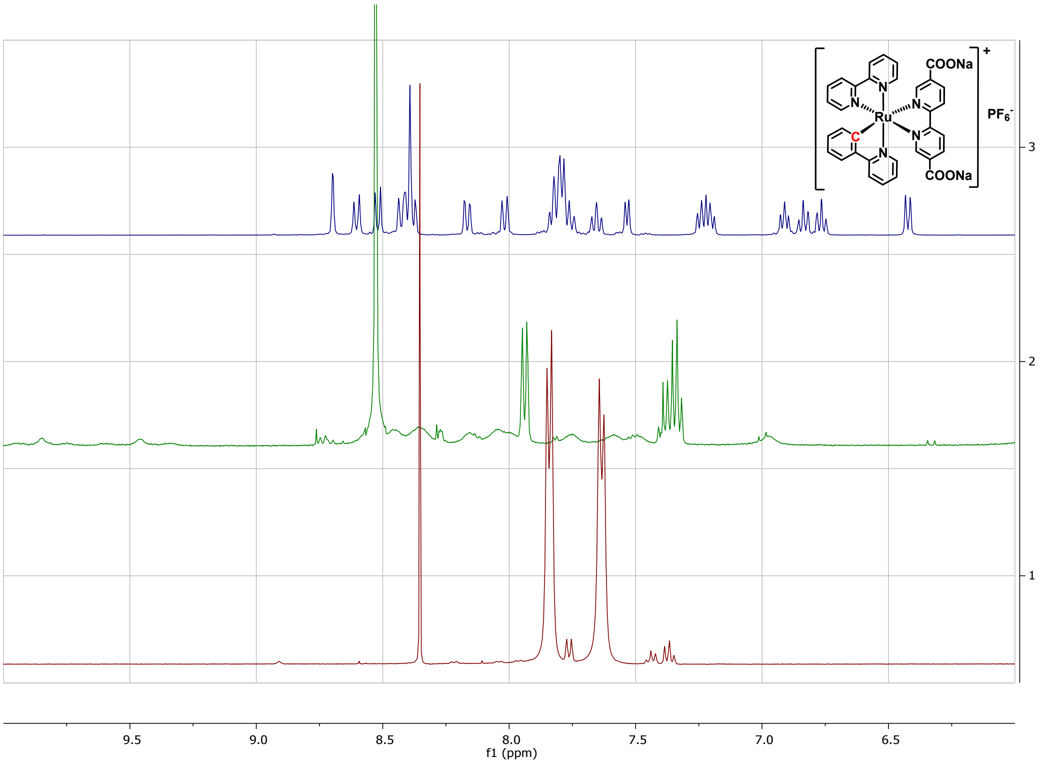

Figure S33. ^{1}**H NMR spectra of 2-Pre digested in 1 M NaOH in D_2O (bottom), redissolved in CD_3OD (middle) and complex 2 in CD_3OD (top).**

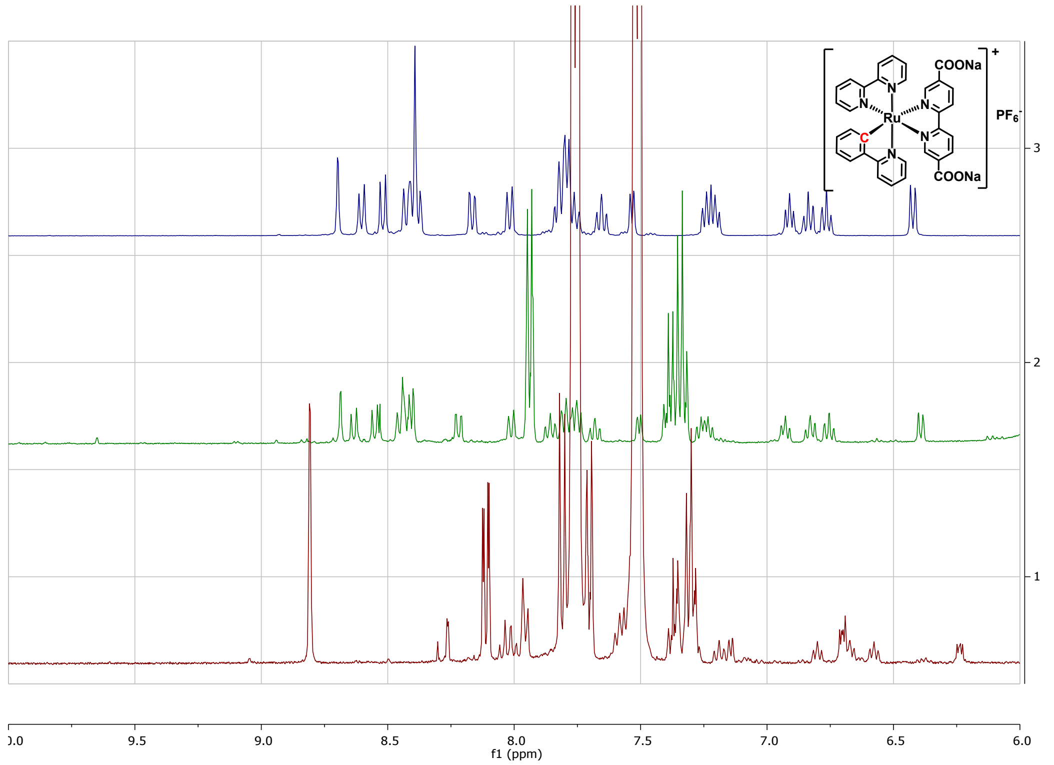

Figure S34. ^{1}H **NMR spectra of 2-Func digested in 1 M NaOH in** D_2O **(bottom), redissolved in** CD_3OD **(middle) and complex 2 in** CD_3OD **(top).**

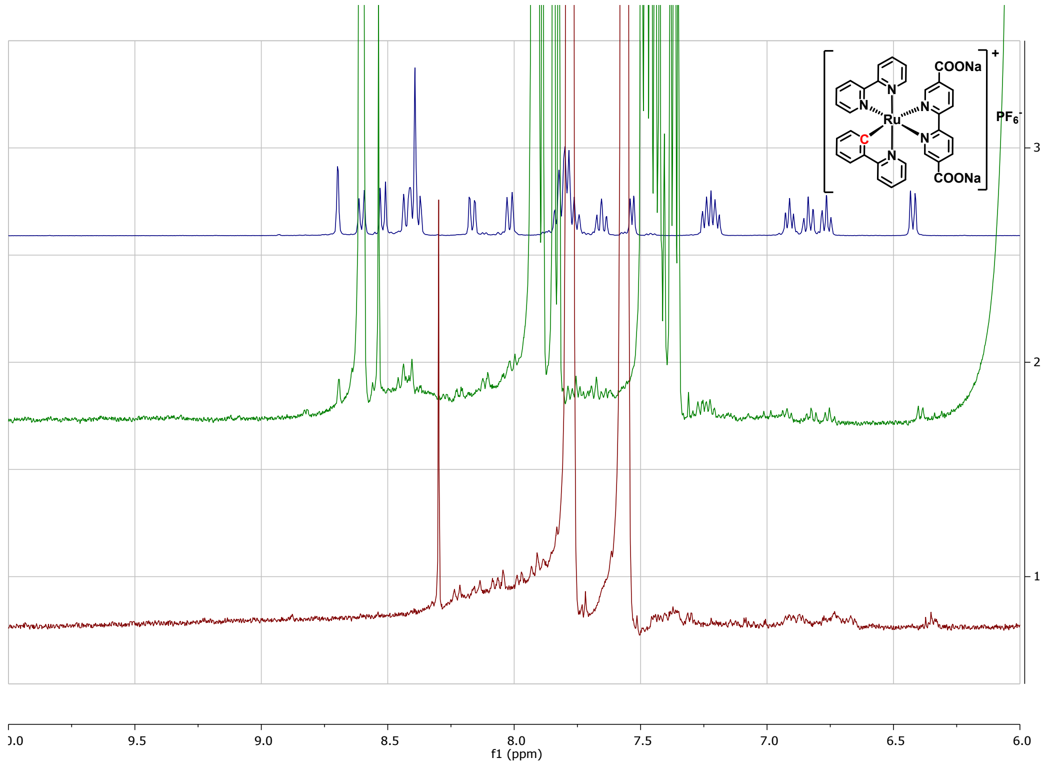

Figure S35. ^{1}H **NMR spectra of 2-Ex digested in 1 M NaOH in** D_2O **(bottom), redissolved in** CD_3OD **(middle) and complex 2 in** CD_3OD **(top).**

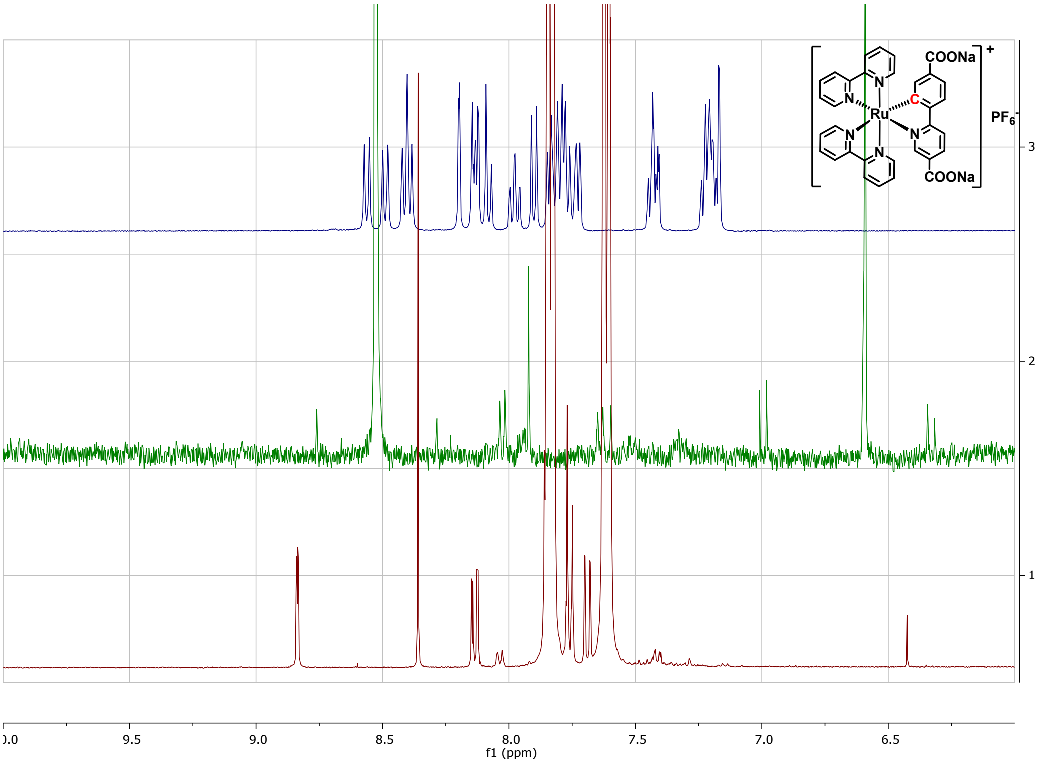

Figure S36. **^{1}H NMR spectra of 3-Pre digested in 1 M NaOH in D_2O (bottom), redissolved in CD_3OD (middle) and complex 3 in CD_3OD (top).**

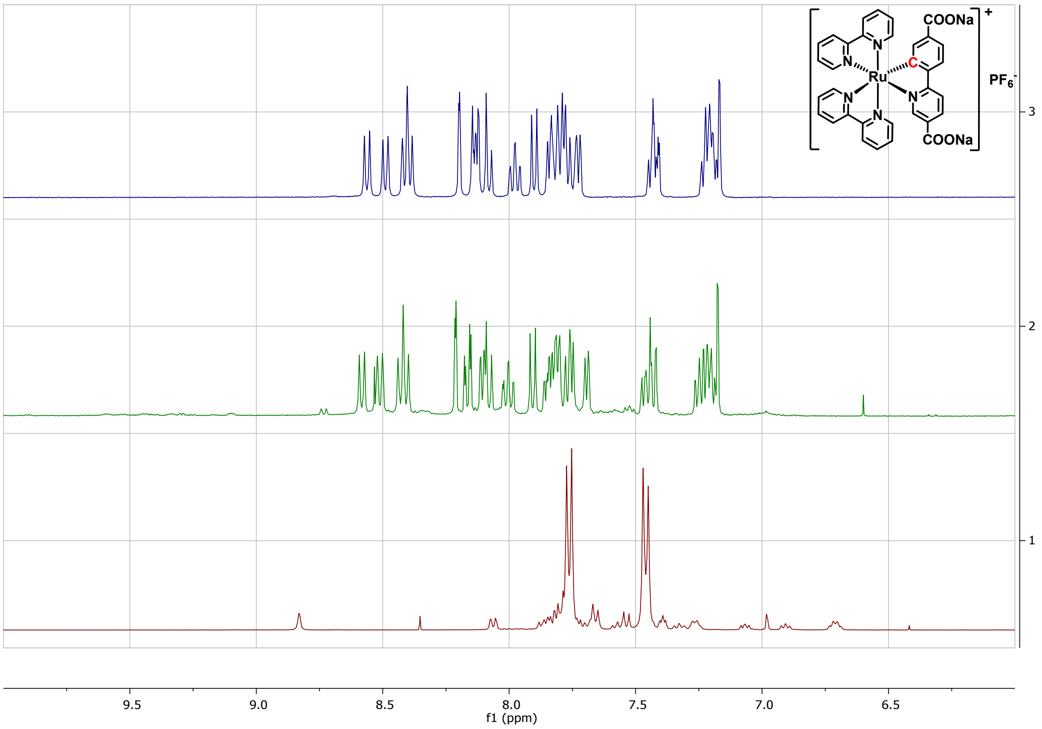

Figure S37. **^{1}H NMR spectra of 3-Ex digested in 1 M NaOH in D_2O (bottom), redissolved in CD_3OD (middle) and complex 3 in CD_3OD (top).**

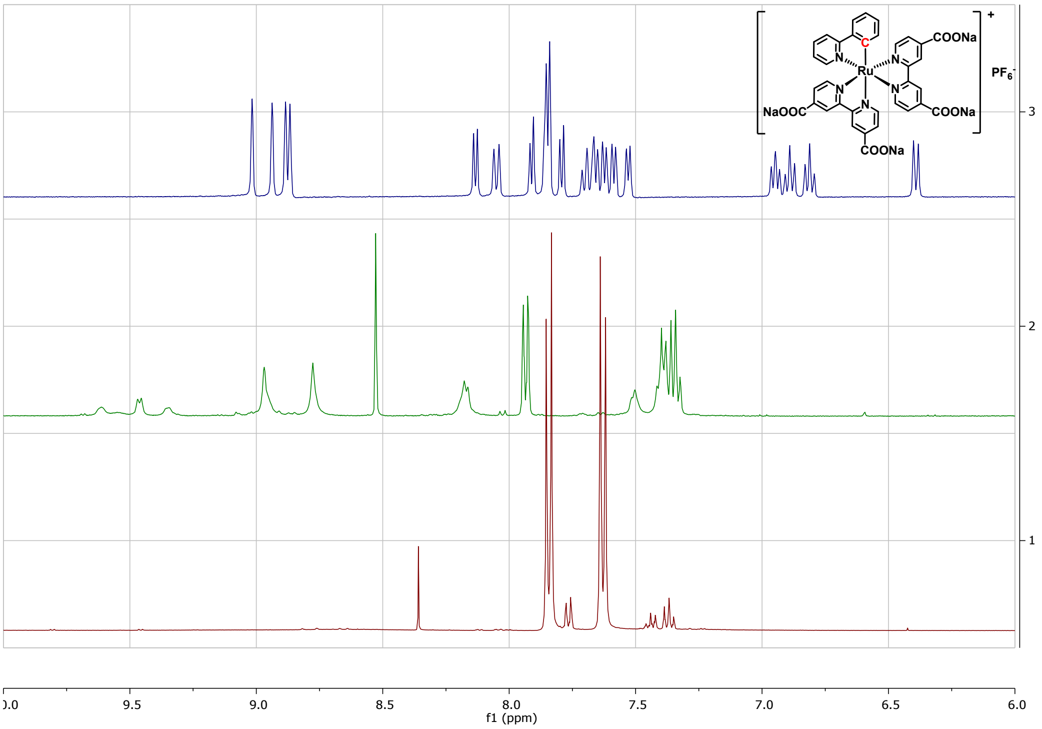

Figure S38. ¹H NMR spectra of 4-Pre digested in 1 M NaOH in D_2O (bottom), redissolved in CD_3OD (middle) and complex 4 in CD_3OD (top).

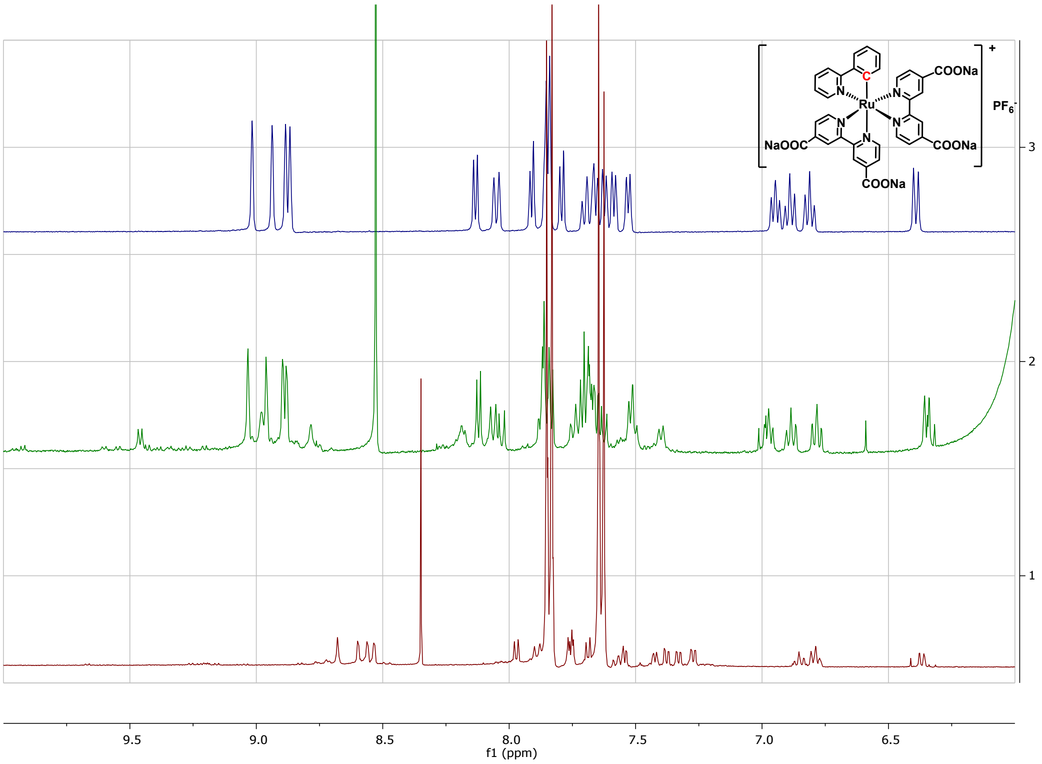

Figure S39. ¹H NMR spectra of 4-Ex digested in 1 M NaOH in D_2O (bottom), redissolved in CD_3OD (middle) and complex 4 in CD_3OD (top).
